# Extraction of Emerging Contaminants from Environmental Waters and Urine by Dispersive Liquid–Liquid Microextraction with Solidification of the Floating Organic Droplet Using Fenchol:Acetic Acid Deep Eutectic Mixtures

**DOI:** 10.1021/acssuschemeng.2c04044

**Published:** 2022-11-16

**Authors:** Cecilia Ortega-Zamora, Gabriel Jiménez-Skrzypek, Javier González-Sálamo, Lucia Mazzapioda, Maria Assunta Navarra, Alessandra Gentili, Javier Hernández-Borges

**Affiliations:** † Departamento de Química, Unidad Departamental de Química Analítica, Facultad de Ciencias, Universidad de La Laguna (ULL), Avda. Astrofísico Fco. Sánchez, s/n, 38206 San Cristóbal de La Laguna, Spain; ‡ Instituto Universitario de Enfermedades Tropicales y Salud Pública de Canarias, Universidad de La Laguna (ULL), Avda. Astrofísico Fco. Sánchez, s/n, 38206 San Cristóbal de La Laguna, Spain; § Department of Chemistry, Sapienza University of Rome, P.le Aldo Moro, 5, 00185 Rome, Italy

**Keywords:** natural deep eutectic solvents, fenchol, emerging contaminants, dispersive liquid−liquid microextraction based on the solidification of the floating organic droplet, high-performance liquid chromatography, mass spectrometry

## Abstract

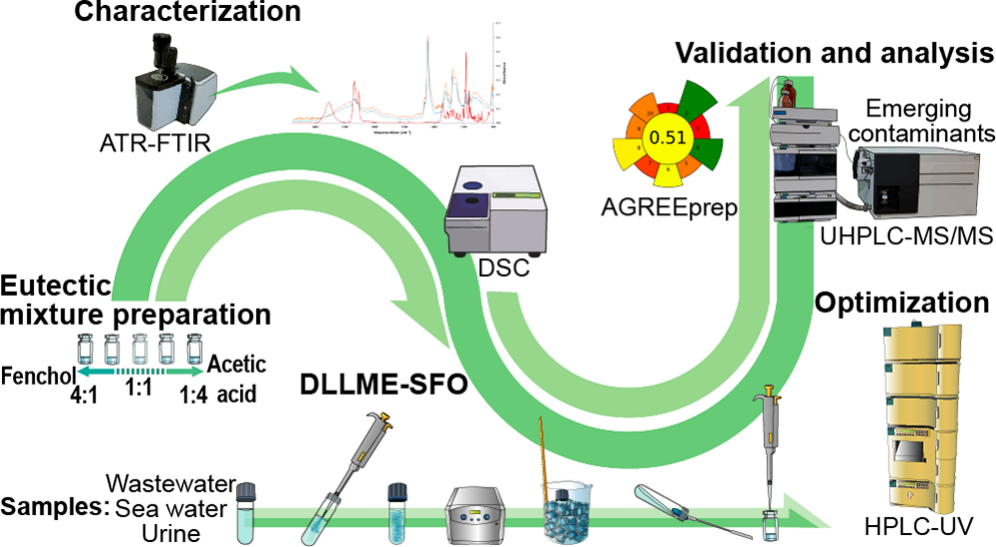

In this work, several eutectic mixtures formed by fenchol and acetic acid at seven molar ratios (between 4:1 and 1:4) were characterized and studied for the first time for their possible application as extraction solvents in dispersive liquid–liquid microextraction based on the solidification of the floating organic droplet (DLLME-SFO). A group of 13 emerging contaminants (gemfibrozil, bisphenol F, bisphenol A, 17β-estradiol, testosterone, estrone, levonorgestrel, 4-*tert*-octylphenol, butyl benzyl phthalate, dibutyl phthalate, 4-octylphenol, 4-nonylphenol, and dihexyl phthalate) was selected and determined by liquid chromatography with ultraviolet and tandem mass spectrometry detection. Among the studied mixtures, only those of 2:1 and 1:1 provided the suitable features from an operational and repeatability point of view, suggesting that several eutectic mixtures of the same components may also provide similar results. Once the extraction conditions of both mixtures were optimized, the method was applied to the extraction of sea water, urine, and wastewater at different concentration levels, allowing the achievement of absolute recovery values between 49 and 100% for most analytes with relative standard deviation values below 19%. In addition, several samples of each type were analyzed, finding bisphenol A and gemfibrozil in some of them. The greenness of the method was also evaluated using the AGREEprep metric. The DLLME-SFO procedure was found to be very simple, quick, and effective and with a good sample throughput.

## Abstract 2

Fenchol:acetic acid eutectic mixtures represent a green and efficient alternative in DLLME-SFO for the extraction of a wide variety of contaminants.

## Introduction

In the last years, important efforts have been made in the analytical chemistry field toward the development of more sustainable analytical methodologies, in an important attempt not only to meet green chemistry principles^1^ maintaining the good performance of the method,^2^ but also to compile with the widely known sustainable development goals.^3^ For this purpose, aspects such as materials hazards, sustainability and renewability, amount of wastes, operator’s safety, energy consumption, sample throughput, automation and miniaturization of the process, among others, are of particular importance, being frequently evaluated using different metrics.^1^

Among the strategies most frequently followed nowadays to achieve such goals, the use of deep eutectic solvents (DESs) combined with liquid-phase microextraction (LPME) techniques can be clearly highlighted as a result of the green properties of many DESs, in particular, the so-called natural DESs (NADESs).^4,5^ DESs have been successfully applied as dispersive liquid–liquid microextraction (DLLME) solvents,^6,7^ as supported liquid membranes for hollow-fiber LPME^8^ as well as solvents for single-drop microextraction,^9^ the three general primary configurations of LPME techniques, which are clearly advantageous to classical liquid–liquid extraction procedures. It should be highlighted that up to now, most of the DESs available are hydrophilic, being those hydrophobic the ideal ones to widen the horizon of greener LPME techniques.

Considering most of the previous applications of DESs in LPME, the most common practice is to select and study in depth a single eutectic mixture for the extraction of a certain group of compounds. On many occasions, such eutectic mixture is assumed to be the one with the lowest melting point or just a eutectic mixture liquid at ambient temperature, which can be conveniently used as the extraction solvent. However, and despite the importance of working with a mixture with the lowest melting point, it is also of high interest to study the full extraction ability of other eutectic mixtures of the same components. This will allow one not only to work under similar or close extraction conditions (which increases the robustness of the method) but also to better understand the chemical interactions involved during the extraction process.

A relatively recent group of hydrophobic natural eutectic mixtures that have been applied with success in LPME, and in particular in DLLME, are those based on the use of menthol,^7,10−12^ a monoterpenoid not classified as hazardous and characterized by its ready biodegradability in the environment. Among the eutectic mixtures that have been proposed, its combination with acetic acid to form quasi-hydrophobic eutectic mixtures results particularly advantageous since this acid can also not be considered a hazardous substance, and it is also cheap and a very frequent reagent in any laboratory. Menthol acts as the hydrogen bond acceptor (HBA), and acetic acid acts as the hydrogen bond donor (HBD). As a result of its high hydrophilicity, acetic acid leaches to the aqueous phase, decreasing its pH value, and leaving to the formation of a menthol-rich phase to which the analytes are extracted.^11,13^ The introduction of alternative monoterpenoids able to form eutectic mixtures could also increase the number of hydrophobic or quasi-hydrophobic DESs available and increase the applicability of LPME techniques to extract different analytes from aqueous samples.

Fenchol (1,3,3-trimethyl-2-norbornanol) is a monoterpene solid at ambient temperature that widely occurs in nature, in particular, in *Cryptomeria japonica* and *Eucalyptus siderophloia*. Its camphoraceous aroma makes it very attractive for its use in the perfume industry. It is a bicyclic molecule with a hydroxyl group at position 2, able to form hydrogen bonds with a suitable HBD. Judging from the ability of similar monoterpenes to form eutectic mixtures, fenchol is a good candidate for obtaining hydrophobic or quasi-hydrophobic NADESs, the use of which may help to extend the application of this type of DESs. However, to the best of our knowledge, this monoterpene has not been proposed yet as part of any eutectic mixture, not even applied as extraction solvent in any miniaturized technique.

Considering the aforementioned, and in order not only to introduce new more sustainable and efficient eutectic mixtures from an extractive point of view but also to obtain more information that allows a better understanding of the chemical interactions involved in the extraction, in this work, different eutectic mixtures that fenchol can form with acetic acid (from 4:1 to 1:4 molar ratios) has been characterized for the first time by studying differential scanning calorimetry (DSC) curves as well as infrared spectra. Furthermore, we also aim at studying the applicability of such eutectic mixtures as solvents in DLLME based on the solidification of the floating organic droplet (DLLME-SFO, which has been found highly advantageous for droplet collection from an operational point of view) for the extraction of a group of 13 emerging contaminants from complex environmental (sea and wastewater) and biological (urine) samples. For this purpose, the parameters affecting the extraction performance in the DLLME-SFO procedure (i.e., sample pH, extraction solvent volume, extraction temperature, NaCl addition, and agitation method and time) have been optimized and the methodology fully validated. The greenness of the method has been also assessed by the AGREEprep metric.^14^ To the best of our knowledge, this work constitutes the first proposal of fenchol as part of a eutectic mixture and the first application of this NADES as LPME solvent (in particular, in DLLME), as well as one of the few studies in which the behavior of different mixtures prepared using different ratios of both components is evaluated.

## Materials and Methods

### Chemicals and Materials

Bisphenol F (BPF, CAS 620-92-8), bisphenol A (BPA, CAS 80-05-7), 17β-estradiol (CAS 50-28-2), testosterone (CAS 58-22-0), estrone (CAS 53-16-7), levonorgestrel (CAS 797-63-7), gemfibrozil (CAS 25812-30-0), 4-*tert*-octylphenol (4-tOP, CAS 140-66-9), butyl benzyl phthalate (BBP, CAS 85-68-7), dibutyl phthalate (DBP, CAS 84-74-2), 4-octylphenol (4-OP, CAS 1806-26-4), 4-nonylphenol (4-NP, CAS 104-40-5), and dihexyl phthalate (DHP, CAS 84-75-3) were used as analytical standards without further purification. All of them have a purity greater than 96% and were acquired from Sigma-Aldrich Chemie (Madrid, Spain) and Dr. Ehrenstorfer (Augsburg, Germany). Their chemical structures and properties are shown in Table S1 of the Supplementary Material. A stock solution of each analyte was prepared at a concentration about 1000 mg/L in methanol or acetonitrile (ACN) and stored at −18 °C in the darkness. All target compounds were mixed in standard solutions at different concentrations, diluted with ACN and stored in the dark at −18 °C.

Fenchol ((1*R*)-*endo*-(+)-fenchyl alcohol, purity 99.3%) was from Sigma-Aldrich Chemie (Schnelldorf, Germany), while glacial acetic acid (purity 100%), hydrochloric acid (purity 37%), sodium hydroxide (purity 99.2%), sodium chloride (purity ≥98%) and ACN of liquid chromatography-mass spectrometry (LC-MS) grade were from VWR International Eurolab (Barcelona, Spain), and ACN LC-MS CHROMASOLV was from Honeywell Riedel-de Haën (Charlotte, NC, USA). Milli-Q water was obtained through the combined use of an Elix Essential tap water purification system and a Milli-Q gradient system A10 from Millipore (Burlington, MA).

Nonvolumetric glassware was cleaned at 550 °C for 4–5 h in a Muffle Carbolite CWF chamber furnace of 13 L capacity and maximum temperature of 1100 °C, whereas for volumetric glassware a sulfuric acid (95% w/w, VWR International Eurolab) solution of Nochromix from Godax Laboratories (Maryland, USA) was used. Besides, in order to prevent the contamination of the samples during their analysis with phthalic acid esters (PAEs), plastic material was avoided whenever possible and blank samples were analyzed with every batch of samples.

### Equipment and Software

A VWR-Hitachi LaChrom Elite 20149 high-performance liquid chromatography (HPLC) system provided with a pump HTA L-2130, an autosampler L-2200, a column oven L-2300, and an ultraviolet (UV) detector L-2400 was used for method optimization. The components of the mobile phase were ACN and water at a constant flow rate of 1.0 mL/min. Separation was carried out using an Eclipse Plus C_18_ column (10 cm × 4.6 mm, 3.5 μm) set at 40 °C after passing through a precolumn (12.5 × 4.6 mm, 5 μm) with the same stationary phase, both acquired from Agilent Technologies (Santa Clara, CA, USA). The elution program was as follows: the initial mobile phase composition was 50:50 (v/v) ACN:water and was held for 4 min, then ACN increased to 70% in 3 min and held for 5 min, finally it was increased to 100% in 6 min and maintained for 10 min before returning to the initial conditions (50:50, v/v). The total run time was 34 min, the injection volume was 20 μL and the wavelength of detection was set at 224 nm for 4-tOP, BBP, DBP, 4-OP, 4-NP, and DHP; 230 nm for BPF, BPA, 17β-estradiol, estrone, and gemfibrozil; 240 nm for levonorgestrel; and 245 nm for testosterone. EZ Chrom Elite software version 3.3.2 SP2 from Agilent Technologies was used in order to control the HPLC-UV system, as well as to integrate and extract the chromatograms.

An Agilent 1260 Infinity II ultra-high-performance liquid chromatography (UHPLC) system equipped with a 1260 Infinity II flexible pump, a 1260 Infinity II vial sampler, an InfinityLab sample thermostat, a 1260 MCT column thermostat, and a tandem mass spectrometry (MS/MS) detector G6470B operated in the dynamic multiple reaction monitoring (MRM) and equipped with an electrospray ionization (ESI) source was used for method validation and real samples analyses. The components of the mobile phase were ACN and water, both containing 0.05% (v/v) of ammonium hydroxide (LC-MS grade, ≥25% in water) from Honeywell Riedel-de Haën, at a constant flow rate of 0.4 mL/min. Separation was performed at 45 °C using an Infinity Lab Poroshell HPH-C_18_ column (100 × 2.1 mm, 2.7 μm) and an Infinity Lab Poroshell HPH-C_18_ precolumn (5 × 2.1 mm, 2.7 μm), both acquired from Agilent Technologies. The elution program started with an initial mobile phase composition of 5:95 (v/v) ACN:water, then ACN increased to 50% in 1 min, finally it was increased to 95% in 2 min and kept at that composition for 5 min before returning to the initial conditions (5:95, v/v). The total run time was 12 min and the injection volume was 5 μL. Regarding the ESI source parameters, the drying gas temperature was set at 210 °C with a flow rate of 7 L/min, the nebulizer pressure was set at 25 psi, and the sheath gas temperature was set at 400 °C with a flow rate of 12 L/min. Capillary voltage was set at 3000 V for both positive and negative polarity, and the nozzle voltage was 0 and −500 V, respectively. Specific conditions regarding *m*/*z* transitions for each of the target compounds, as well as other parameters are specified in Table S2 of the Supplementary Material. The UHPLC-MS/MS system was controlled using Agilent MassHunter Workstation Data LC/MS Data Acquisition software (version 10.1, build 10.1.67), while Agilent MassHunter Workstation Qualitative Analysis (version 10.0, build 10.0.10305.0) was used for extracting chromatograms and Agilent MassHunter Workstation Quantitative Analysis (version 10.1, build 10.1.733.0) was used for integration and data extraction.

An analytical balance 224i-1S from Sartorius (Goettingen, Germany), a magnetic agitator and heater from IKA RET basic (Deutschland, Germany), and a Mega Star 3.0R centrifuge from VWR International Eurolab were used. A Rear Top vortex and an Ultrasonic Cleaner USC-600T working at 120 W and 45 kHz, both from VWR International Eurolab, were used for the optimization of the dispersion of the extractant solvent in the sample. The pH and conductivity were measured using a Five Easy Plus pH/mV meter from Mettler Toledo (Columbus, OH, USA) and a CM 35+ conductivity meter from Crison (Barcelona, Spain), respectively.

### Eutectic Mixtures Characterization

A Cary 630 Fourier Transform infrared (FTIR) spectrometer coupled to a single reflection diamond attenuated total reflectance (ATR) sampling module from Agilent Technologies was used for IR measurements. It was equipped with a ZnSe beamsplitter and a 1.3 mm diameter thermoelectrically cooled deuterium triglycine sulfate detector. Each sample ATR-FTIR spectrum was obtained at a resolution of 8 cm^–1^ with 64 scans, and in a wavenumber range between 4000 and 600 cm^–1^. Agilent MicroLab PC software was used for data acquisition and analysis.

Thermal analyses were conducted using a DSC821 instrument (Mettler Toledo, Zaventem, Belgium). DSC measurements were carried out under nitrogen atmosphere at a flow of 60 mL/min following a cooling and heating procedure from room temperature to −120 °C at a rate of −10 °C/min, keeping the sample at −120 °C for 5 min, and increasing the temperature from −120 to 100 °C at a rate of 10 °C/min. Transition temperatures were detected and reported in the paper, by the on-set values corresponding to each thermal process.

### Samples

The optimized microextraction procedure was validated for wastewater, sea water, and human urine (the last of them was obtained from healthy volunteers). The three different matrices were filtered before their use through polyvinylidene fluoride membrane filters of 0.22 μm pore size. In addition to the matrices used with validation purposes, five more wastewater samples collected at different wastewater treatment plants (WWTPs) in Tenerife (Canary Islands, Spain) and one more sea water sample collected at one of the beaches of the island of La Palma (Canary Islands, Spain) were also analyzed. Table S3 of the Supplementary Material shows pH and conductivity data of all samples.

### Natural Eutectic Mixtures Preparation

The prepared eutectic mixtures were composed of fenchol as HBA and acetic acid as HBD in molar ratios between 4:1 and 1:4. The appropriate amounts of each of the components were introduced into clear glass vials with a screw cap and mixed under constant stirring at 400 rpm for 1 min at 80 °C, obtaining a clear and colorless liquid, which was cooled down to room temperature before use. Both compounds were used without further purification; fenchol was kept in a desiccator and glacial acetic acid was taken from a new commercial bottle, just opened for this purpose. Although acetic acid may be hygroscopic, no hydration problems occurred due to the simplicity and the short period of time that the developed methodology entailed. In addition, the eutectic mixtures prepared can be used for several days since they remain liquid at room temperature and do not crystallize or hydrate in the following days if they are kept in a desiccator.

### Extraction Procedure

Twenty milliliters of the sample previously adjusted to pH 6.0 using 0.1 M NaOH or 0.1 M HCl solutions and at 25 °C were placed in a conical bottom glass centrifuge tube. Later, 100 or 90 μL of the eutectic mixtures 2:1 and 1:1, respectively, were quickly added and the sample was manually stirred vigorously for 1 min, obtaining a cloudy solution. Then, the aqueous phase was separated from the organic phase by centrifugation for 10 min at 2500 rpm (1363 × *g*). Afterward, the tubes were placed in an ice bath for 5 min in order to obtain a solidified droplet of the eutectic mixture at the top of the solution. Finally, the solidified droplet was removed with a stainless-steel spatula, transferred to a glass vial, and dissolved in 2567 and 2310 μL of a mixture ACN:H_2_O (50:50, v/v) for 2:1 and 1:1 molar ratios, respectively. The homogeneous and transparent solution obtained was injected into the HPLC-UV system for the extraction method optimization (20 μL) and into the UHPLC-MS/MS system for method validation and real samples analysis (5 μL).

It should be noted that in the case of urine and wastewater samples, the agitation should not produce froth because it prevents the subsequent formation of the solidified droplet (a cloudy solution can still be observed after centrifugation).

## Results and DICUSSION

### HPLC-UV and UHPLC-MS/MS Analysis

In this work, 13 emerging contaminants, including 5 phenols, 4 estrogens, 1 pharmaceutical, and 3 PAEs, were selected as model analytes and separated by LC under the conditions previously described in the Experimental Section. HPLC-UV was used for method optimization (a representative chromatogram is shown in Figure S1 of the Supplementary Material), while UHPLC-MS/MS was used for method validation and real samples analyses (a representative chromatogram is shown in Figure S2 of the Supplementary Material). Among the selected PAEs, there are some of those for which the EU has established tolerable daily intake values (i.e., BBP and DBP).^15^ Estrone and 17β-estradiol are endogenous natural estrogens (endoestrogens), which have been found in human urine and environmental waters,^16^ as well as the target phenols (i.e., BPA, BPF, 4-tOP, 4-OP, and 4-NP).^17,18^ These compounds are considered as endocrine disruptors together with testosterone and levonorgestrel (the biologically active form of norgestrel), which is why some of them have been included in the preliminary list of priority substances,^19,20^ in particular, 17β-estradiol and testosterone have been prohibited by the EU since 1981 through some directives to be used as growth promoters in farm animals.^21−23^

Before injecting the eutectic mixtures into the LC system, they must be suitably dissolved in a mixture ACN:water 50:50 (v/v). For this purpose, different volumes of the eutectic mixtures (10, 15, 20, 30, and 35 μL) were tested to achieve a final volume of 400 μL. For the 2:1 and 1:1 molar ratios of fenchol:acetic acid mixtures, it was found that 15 μL dissolved correctly in 385 μL of the ACN:water mixture, while for the 1:2 molar ratio mixture, 30 μL dissolved in 370 μL of the ACN:water mixture. The necessary volumes to dissolve the other mixtures were not tested because they were not useful for the DLLME-SFO procedure, as it will be explained later. These proportions were the ones used in later analyses for the dissolution of the eutectic mixtures before their injection in the HPLC-UV or UHPLC-MS/MS.

The repeatability of the injection and analyte separation in the HPLC-UV system was studied by injecting three different concentrations (100, 500, and 1000 μg/L) five times each (*n* = 5) on three consecutive days. The data resulting from these studies showed an acceptable intraday and interday precision as can be observed in Tables S4 and S5 of the Supplementary Material (relative standard deviation (RSD) values, below 4.3 and 7.3% in the same day and between days, respectively, for the peak areas; and below 0.12 and 0.14% for the retention times, respectively). The repeatability study was also carried out at 10, 50, and 100 μg/L in the UHPLC-MS/MS system. RSD values were below 11.6 and 17.9% in the same day and between days, respectively, for the peak areas; and below 0.15 and 0.37% for the retention times, respectively, (see Tables S6 and S7 of the Supplementary Material). Subsequently, an instrumental calibration study was carried out in the two chromatographic systems by injecting eight different concentrations levels of each analyte (*n* = 8). In both cases, determination coefficient (*R*^2^) values higher than 0.991 were obtained, which indicates that a good linearity was acquired in the range of concentrations studied. Tables S8 and S9 of the Supplementary Material show these data together with the slopes and intercepts values, their confidence intervals as well as the error of the estimate. Concerning the lowest calibration levels, they were in the range 25–38 μg/L in the HPLC-UV system and between 1 and 1.5 μg/L in the UHPLC-MS/MS system.

### Characterization of the Eutectic Mixtures

The characterization of the seven eutectic mixtures was carried out by means of ATR-FTIR measurements. Figure S3 of the Supplementary Material shows the ATR-FTIR spectra of fenchol, acetic acid and all the mixtures at different molar ratios. In them, it can be observed that fenchol presents a characteristic band at 3365 cm^–1^ corresponding to the hydroxyl group, as well as at 2855–2970 cm^–1^ due to the sp^3^ C–H stretch. As the proportion of fenchol decreases and the molar ratio of the two components approaches to 1:1, the band corresponding to the hydroxyl group widens and shifts slightly to higher values. Subsequently, when the proportion of acetic acid continues to increase, both the representative band of the hydroxyl group of fenchol and that of the sp^3^ C–H bonds cannot be easily distinguished. On the other hand, in the acetic acid spectrum, a characteristic band of the stretching movement of the carbonyl group C=O is observed at 1707 cm^–1^, which decreases in intensity as the proportion of acetic in the mixture decreases. In addition, it can be seen that the C–O–H bond bending band of fenchol at 1062 cm^–1^ and the C–O bond tension band of acetic acid at 1285 cm^–1^could be identified. The spectral changes when varying the molar proportions in the mixture indicate the presence of interactions between the two initial components. This fact is similar to that previously found between l-menthol and acetic acid eutectic mixtures.^11^

In addition, DSC curves were obtained and shown in Figure S4 of the Supplementary Material. Figure S4A displays the DSC curves for pure acetic acid, the 1:1 mixture and the mixtures with acetic acid as the main component; here, the eutectic (−27.39 °C) and melting point (16.91 °C) of pure acetic acid can be seen.^24^ From all the acetic acid concentrated mixtures, a glass transition peak appears around −90 °C, which means that all these compositions present an amorphous structure which then transforms into a more ordered one upon heating, as visible from the exothermic peak at about −32 °C. For the 1:2 mixture, two exothermic peaks are observed due to the so-called cold crystallization process. In all samples, the recrystallization process is followed by two melting processes, one set in a very narrow temperature range (−11 to −14 °C) and the other observed at small higher temperatures. The first melting transition could be attributed to the eutectic composition, and its intensity increases when the amount of acetic acid decreases. On the contrary, the intensity of the second melting process increases with the amount of acetic acid. This peak can certainly be attributed to the melting point of acetic acid. Figure S4B reports the curves for pure fenchol and the mixtures with fenchol as the main component. For the mixture at a 2:1 molar ratio, a single peak is observed, corresponding to the glass transition temperature. As it has been previously reported,^25^ this mixture can be considered as a low transition temperature mixture because no crystallization or melting phenomena can be clearly observed. In the 3:1 and 4:1 mixtures, very small peaks related to eutectic points of acetic acid and the mixtures are present at about −25 and −15 °C, respectively; moreover, a new bigger peak appears at higher temperature in the fenchol-concentrated mixtures: at 12.95 °C for the 3:1 mixture and 23.54 °C for the 4:1 mixture. Indeed, the temperature and intensity associated to this peak increase as the amount of fenchol increases up to its characteristic melting point.

Judging from these results, and as it will be later shown, only some of the mixtures could be used in DLLME-SFO from an operational point of view.

### Optimization of the DLLME-SFO Procedure

Once the eutectic mixtures were prepared and characterized, they were used as extraction solvents in DLLME-SFO for the determination of the 13 target compounds in human urine, wastewater, and sea water samples. This microextraction technique was selected since the physicochemical characteristics of eutectic solvents have previously shown very promising results in DLLME procedures.^26^ However, on some occasions, the removal of the droplet is really difficult and irrepeatable. In such cases, the solidification of the floating organic droplet makes its collection possible as well as faster, simpler, and easier, ensuring its extraction in its entirety from the sample solution. As a consequence, and in order to obtain optimal extraction conditions, sample pH, eutectic mixture volume, extraction temperature, agitation type and time, and NaCl effect were studied using 20 mL of Milli-Q water. Absolute recovery values obtained from the spiked samples before and after the extraction procedure at a concentration of 500 μg/L of all the target analytes were evaluated for each optimized parameter.

### Eutectic Mixture Composition

As previously indicated, in the literature, a large number of works can be found that only study and use a single eutectic mixture for the extraction of a certain group of compounds. However, it is also of high interest to study the full extraction ability of other eutectic mixtures of the same compounds. This will allow not only to work under similar or close extraction conditions (which increases the robustness of the method) but also to better understand the chemical interactions involved during the extraction process. In this sense, after synthesizing the seven mixtures of fenchol and acetic acid at different molar ratios (between 4:1 and 1:4) as it was previously described, they were cooled to room temperature, putting them in the fridge and freezer at temperatures between 4 and −18 °C. When the samples reached these temperatures, they were left for at least 1 h to check if they experienced any change in their state. It was observed that those mixtures with a higher proportion of one of the components solidified at −18 °C (i.e., 4:1, 3:1, 1:3, and 1:4 molar ratios) as DSC measurements have shown, since they are far from being the eutectic mixture with the lowest melting temperature. Since mixtures 2:1, 1:1, and 1:2 did not solidify because they showed the lowest melting temperatures, as it was seen in the DSC plots, they were selected for further studies.

### Sample pH Effect

**Figure 1:**
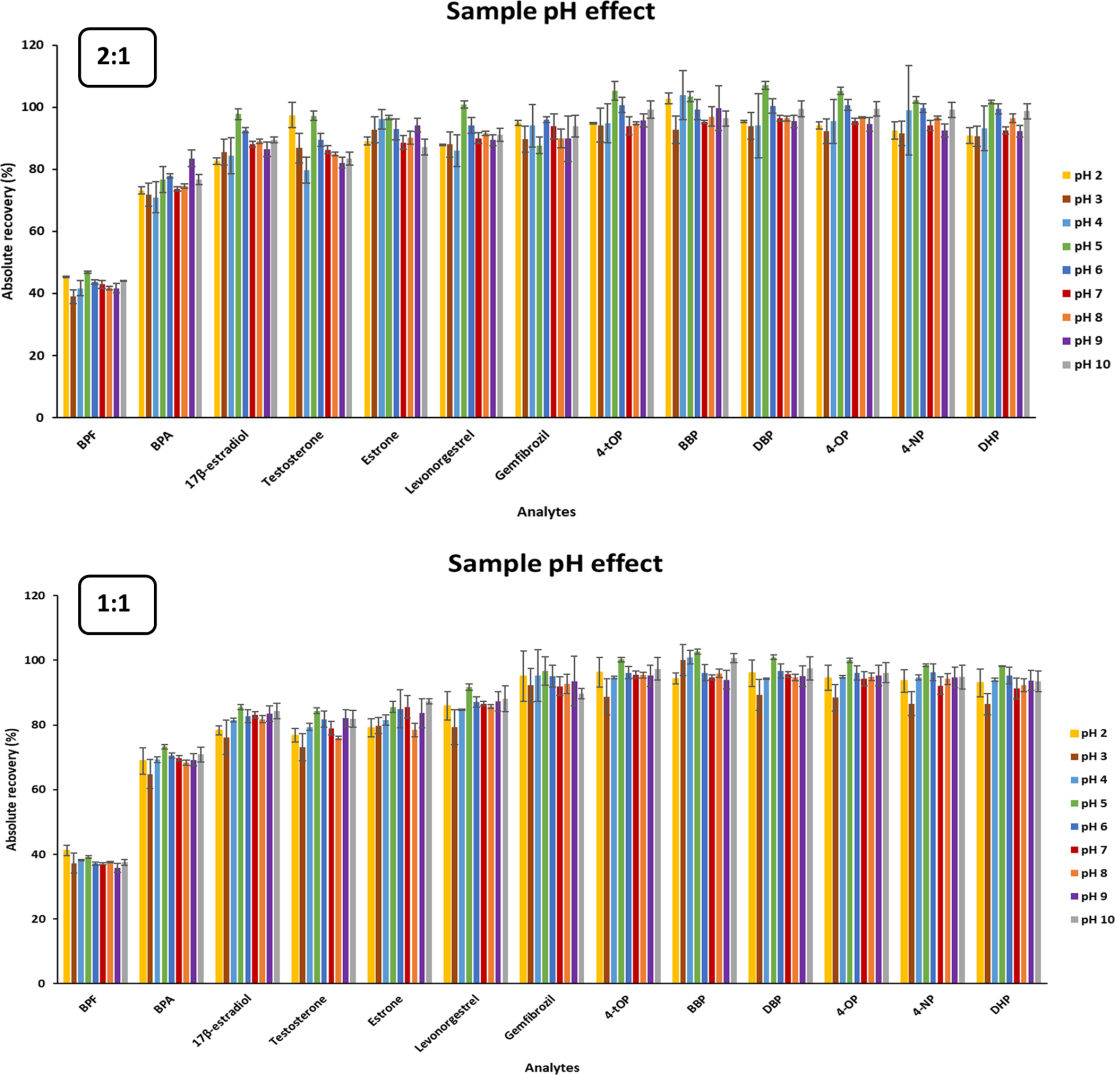
Effect of the sample pH on the absolute recovery values of the selected emerging contaminants using 2:1 and 1:1 molar ratio mixtures of fenchol:acetic acid as DLLME-SFO solvent. Extraction conditions: 20 mL of spiked Milli-Q water at 0.50 mg/L, except for 17β-estradiol and estrone which was 0.75 mg/L, at 25 °C, 100 μL of eutectic mixture, and manual agitation for 1 min.

The assessment of the effect of sample pH was performed adjusting 20 mL of Milli-Q water at pH values between 2 and 10 with the NaOH and HCl solutions indicated in the Experimental Section. This parameter was studied for the eutectic mixtures fenchol:acetic acid at 2:1, 1:1, and 1:2 molar ratios; three extractions were carried out for each pH value. As can be seen in Figure 1, the pH of the sample does not affect the extraction of the analytes when the eutectic mixtures at 2:1 and 1:1 molar ratios are used. Regarding the 1:2 mixture (see Figure S5 of the Supplementary Material), a greater variability in the recovery values was obtained: 71–107% for 2:1 molar ratio, 65–103% for 1:1 molar ratio, and 48–114% for 1:2 molar ratio, except for the BPF, which showed lower recovery values). This may be due to the fact that the hydrophilic nature of acetic acid causes a partial leaching into the aqueous sample as previously reported for other eutectic mixture of acetic acid.^11^ For this reason, the optimization study was not continued with this proportion of the two components (1:2 mixture), since the other two provided higher recovery values also with a higher repeatability (RSD values for the 1:2 mixture was in the range 1–16%). Concerning the pH of the sample, and despite it did not influence the extraction, it was decided to maintain it at a constant value (pH 6.0), paying also particular attention to the fact that the p*K*_a_ value of acetic acid is ∼4.75 at 25 °C in order to ensure that all acetic acid was in the same form (deprotonated).

It is also important to mention that, as a consequence of the leaching of acetic acid, it would be expected that fenchol was the main responsible for extracting the analytes after its proper dissolution. However, lower recovery values were obtained for all the analytes after an amount of fenchol (no acetic acid was added) equivalent to that in the volume of the extraction solvent used was added to Milli-Q water at pH 6.0 (50–78 and 33% for BPF); besides, a water sample should be heated around 45 °C (the melting temperature of fenchol), which would require higher energy consumption in each extraction, which it is not necessary with the direct use of the already prepared eutectic mixtures.

### Extraction Solvent Volume Effect

Subsequently, the effect of the volume of 2:1 and 1:1 eutectic mixtures on the recovery values was studied. For this purpose, different volumes (70, 80, 90, 100, 110, and 120 μL) were added to the sample solution at pH 6.0 (three extractions were carried out at each volume), and the solidified droplet was dissolved with an appropriate volume of a mixture ACN:water (50:50, v/v). Figure S6 of the Supplementary Material shows the variation of the absolute recovery values with the solvent volume for each ratio. It can be observed that slightly higher recovery values were obtained with 100 μL of the eutectic mixture at 2:1 molar ratio (especially for testosterone), while with the 1:1 molar ratio, significant differences were not perceived between 90 and 100 μL; therefore, 90 μL were chosen for further studies in order to consume a lower amount of the solvent.

### Extraction Temperature Effect

To test the temperature effect, triplicate extractions were carried out at 20, 25, and 30 °C. Figure S7 of the Supplementary Material shows the results of this study. Given that higher recovery values were obtained for most of the analytes when the sample solution was set at 25 °C, this temperature was selected for further analysis for both molar ratios, though it should be indicated that no significant changes were found between the different temperatures.

### Agitation Method and Time Effect

In any dispersive version of an extraction methodology, it is essential to ensure a good dispersion of the solvent in the sample matrix in order to improve the extraction efficiency and to accelerate the process. In this case, although good recovery values were achieved by applying manual agitation for 1 min, vortex and ultrasound were also tested, and the agitation time was also assessed for all the agitation modes. In this sense, the three methods were tested at 30 s, 1 min, and 2 min. The extractions were carried out in triplicate and at the previously optimized conditions (20 mL of Milli-Q water at pH 6.0 and 25 °C, and with 90 or 100 μL of the eutectic mixture at 1:1 and 2:1 molar ratio, respectively). From the results obtained (data not shown), manual agitation for 1 min provided the highest absolute recovery values, in the range 69–100% for 1:1 molar ratio and 78–101% for 2:1 molar ratio (except for BPF for which the recovery values were 34 and 44%, respectively). These data were higher than those obtained with the other extraction times and agitation methods evaluated (in such cases recovery values were in the range 2–96% for 1:1 molar ratio and 3–105% for 2:1 molar ratio). Therefore, manual agitation for 1 min was used in further studies.

### Salting-Out Effect

**Figure 2:**
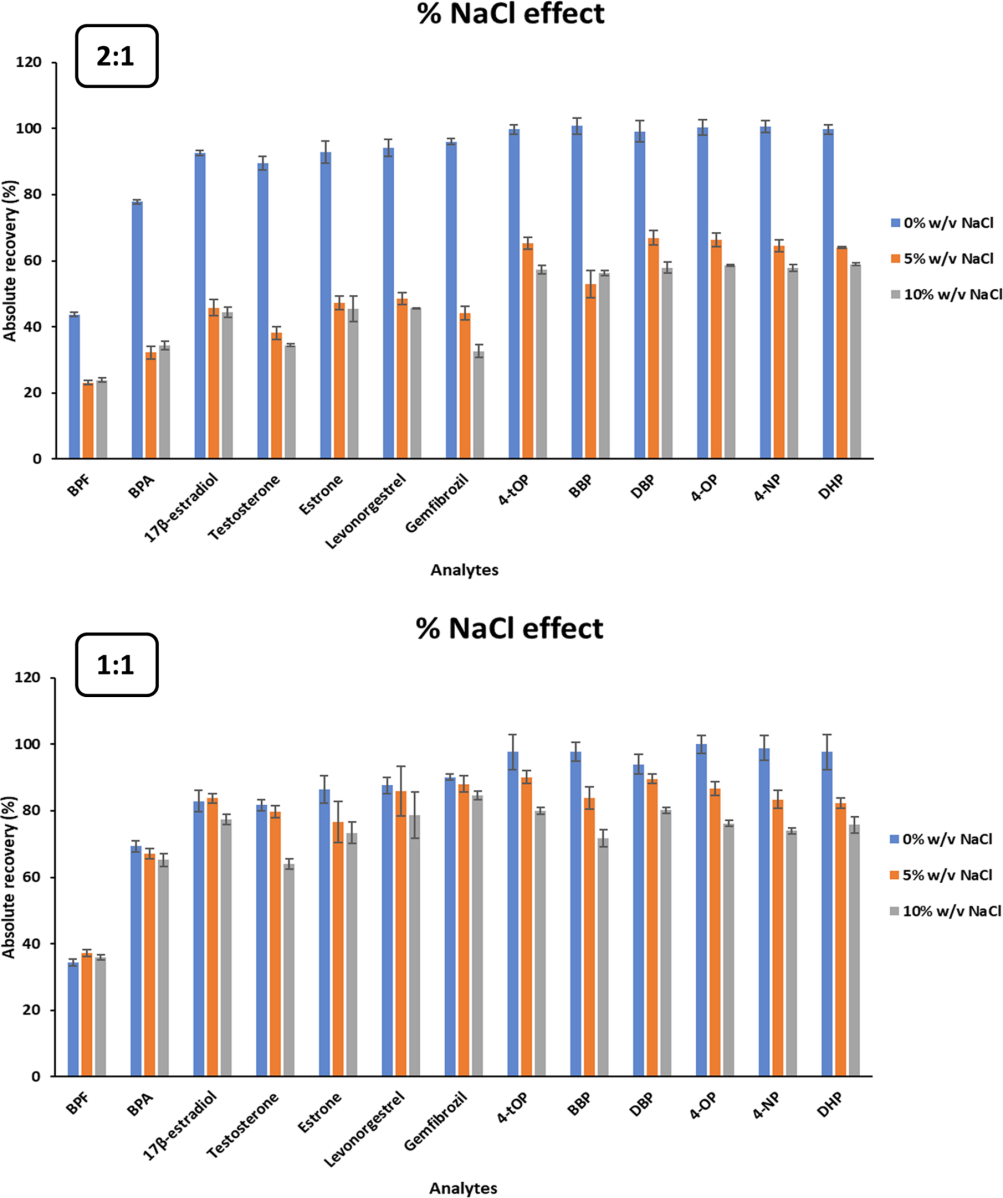
Effect of the addition of NaCl to the sample on the absolute recovery values of the selected emerging contaminants using 2:1 and 1:1 molar ratio mixtures of fenchol:acetic acid as DLLME-SFO solvent. Extraction conditions: 20 mL of spiked Milli-Q water at 0.50 mg/L, except for 17β-estradiol and estrone which was 0.75 mg/L, at pH 6.0 and 25 °C, 100 μL of eutectic mixture at 2:1 molar ratio and 90 μL for the one at 1:1 molar ratio, and manual agitation for 1 min.

The salting-out effect was also studied since the addition of NaCl to the aqueous phase can modify the ionic strength of the sample solution and, as a consequence, affect the extraction procedure. For this reason, extractions were performed by adding 0, 5, and 10% (w/v) of NaCl to the sample solution. The results obtained (see Figure 2) showed that the addition of this salt negatively affected the extraction of the target compounds, especially with the extraction solvent at 2:1 molar ratio. According to these data, no NaCl was added during the DLLME-SFO procedure.

### Method Validation

After having established the conditions that allow the achievement of the highest extraction efficiency, the DLLME-SFO method was applied to the extraction of the target compounds from human urine, sea water, and wastewater samples, using in this case an UHPLC-MS/MS system in order to improve the sensitivity of the method and to allow unequivocal confirmation of the analytes. For method validation, SANTE Guidelines from the European Commission^27^ have been taken as a reference to assess the trueness, repeatability, linearity, sensitivity, and matrix effect (ME). Thus, each matrix was spiked with the target analytes at three different concentration levels and five consecutive extractions were carried out with each one with the aim of studying the trueness of the developed methodology. Recovery values were calculated considering the peak area of each analyte of a blank sample spiked before extraction and the one obtained from a blank sample spiked in the extract obtained after extraction. As can be seen in Table 1, absolute recovery values in the two eutectic mixtures considered were very similar (between 51 and 100% for the 2:1 molar ratio and between 49 and 99% for the 1:1, except for BPF for which they were in the range 43–54 and 39–53%, respectively), with RSD values below 19% in both cases.

**Table 1:** Absolute Recovery and RSD (in Parentheses) Values after the Application of the DLLME-SFO-UHPLC-MS/MS Method to the Extraction of the Target Analytes from Human Urine, Wastewater, and Sea Water (*n* = 5 at each Spiking Level) Using 2:1 and 1:1 Fenchol:Acetic Acid Eutectic Mixtures

analytes	molar ratio eutectic mixture (fenchol:acetic acid)	sample	level 1^a^	level 2^a^	level 3^a^
			recovery % (RSD %)	recovery % (RSD %)	recovery % (RSD %)
BPF	2:1	urine	49 (4)	54 (3)	45 (5)
		wastewater	43 (7)	49 (7)	47 (5)
		sea water	47 (5)	51 (5)	49 (5)
	1:1	urine	39 (17)	48 (8)	49 (8)
		wastewater	48 (8)	49 (11)	53 (8)
		sea water	47 (13)	45 (6)	49 (8)
BPA	2:1	urine	71 (12)	100 (11)	70 (4)
		wastewater	85 (14)	94 (5)	85 (15)
		sea water	65 (7)	80 (14)	74 (10)
	1:1	urine	70 (18)	84 (4)	60 (8)
		wastewater	86 (2)	92 (16)	79 (3)
		sea water	52 (4)	49 (5)	52 (8)
17β-estradiol	2:1	urine	57 (13)	78 (7)	51 (12)
		wastewater	89 (13)	74 (6)	65 (6)
		sea water	53 (8)	53 (6)	59 (9)
	1:1	urine	67 (4)	74 (3)	68 (6)
		wastewater	71 (4)	62 (2)	53 (10)
		sea water	75 (3)	72 (7)	64 (7)
testosterone	2:1	urine	66 (13)	78 (10)	69 (9)
		wastewater	60 (16)	67 (5)	61 (9)
		sea water	59 (11)	54 (6)	58 (9)
	1:1	urine	49 (13)	59 (5)	52 (11)
		wastewater	53 (13)	49 (8)	53 (8)
		sea water	59 (8)	52 (6)	53 (7)
estrone	2:1	urine	80 (12)	85 (10)	70 (2)
		wastewater	85 (5)	76 (6)	64 (5)
		sea water	61 (5)	59 (8)	57 (10)
	1:1	urine	64 (16)	74 (3)	78 (6)
		wastewater	85 (10)	69 (4)	73 (5)
		sea water	85 (7)	93 (6)	64 (13)
levonorgestrel	2:1	urine	76 (15)	77 (4)	51 (7)
		wastewater	87 (5)	73 (6)	62 (2)
		sea water	51 (4)	53 (7)	59 (9)
	1:1	urine	70 (6)	53 (3)	50 (8)
		wastewater	89 (7)	73 (3)	66 (7)
		sea water	75 (7)	71 (3)	68 (8)
gemfibrozil	2:1	urine	81 (14)	91 (6)	72 (10)
		wastewater	81 (6)	83 (6)	90 (11)
		sea water	63 (11)	59 (6)	59 (12)
	1:1	urine	88 (5)	74 (6)	75 (6)
		wastewater	84 (4)	99 (2)	80 (2)
		sea water	83 (5)	84 (14)	81 (10)
4-tOP	2:1	urine	64 (2)	86 (8)	71 (3)
		wastewater	67 (7)	76 (6)	68 (7)
		sea water	62 (3)	53 (7)	61 (8)
	1:1	urine	61 (6)	70 (4)	73 (7)
		wastewater	81 (10)	69 (6)	78 (9)
		sea water	74 (16)	71 (5)	74 (9)
BBP	2:1	urine	63 (14)	80 (8)	100 (10)
		wastewater	75 (2)	69 (9)	65 (6)
		sea water	72 (8)	75 (12)	70 (4)
	1:1	urine	77 (9)	82 (2)	72 (5)
		wastewater	72 (19)	61 (4)	76 (8)
		sea water	68 (14)	69 (6)	69 (8)
DBP	2:1	urine	82 (7)	85 (7)	63 (6)
		wastewater	84 (6)	80 (5)	73 (3)
		sea water	57 (10)	54 (7)	62 (8)
	1:1	urine	82 (11)	76 (3)	68 (6)
		wastewater	81 (7)	67 (4)	78 (6)
		sea water	78 (7)	76 (4)	76 (9)
4-OP	2:1	urine	61 (9)	81 (6)	62 (6)
		wastewater	93 (3)	78 (5)	74 (4)
		sea water	58 (13)	52 (7)	60 (7)
	1:1	urine	79 (13)	70 (4)	63 (5)
		wastewater	85 (9)	67 (6)	76 (9)
		sea water	80 (6)	74 (4)	73 (9)
4-NP	2:1	urine	61 (6)	78 (11)	63 (10)
		wastewater	93 (3)	77 (5)	74 (2)
		sea water	62 (6)	55 (5)	58 (7)
	1:1	urine	71 (12)	68 (4)	62 (5)
		wastewater	76 (4)	65 (7)	74 (10)
		sea water	77 (11)	72 (3)	71 (9)
DHP	2:1	urine	76 (12)	68 (5)	66 (9)
		wastewater	87 (11)	78 (5)	73 (9)
		sea water	53 (3)	51 (8)	59 (8)
	1:1	urine	81 (11)	74 (4)	66 (5)
		wastewater	91 (9)	68 (7)	74 (9)
		sea water	80 (7)	77 (4)	73 (9)

a The sample concentrations were as follows: level 1: 0.13 μg/L; level 2: 7 μg/L, and level 3: 13 μg/L for all the analytes except for 17β-estradiol and estrone (level 1: 0.20 μg/L, level 2: 10 μg/L, and level 3: 20 μg/L).

Subsequently, a matrix-matched calibration was carried out at eight concentration levels for both eutectic mixtures in the three types of samples (matrices were treated as a blank and spiked after the DLLME-SFO procedure). Procedural blanks and nonspiked samples were also analyzed, and if any of the target analytes was found in them (in particular, any PAEs, which are the ones that could also be present in the laboratory^28^), the signal was subtracted in successive calculations. The studied linear range, the equations of the calibration curves with the corresponding confidence intervals of the slope and intercept, as well as the error of the estimate, the R^2^ values, and the limits of quantification (LOQs) of the method are shown in Table S10 of the Supplementary Material. In all cases a good linearity was achieved, with *R*^2^ values above 0.991. Regarding sensitivity, the LOQs of the method were estimated from the lowest matrix-matched calibration levels for each analyte and matrix. All these values were experimentally checked by injecting the final extract of a blank sample spiked with the analytes at the LODs and LOQs concentration before extraction.

**Figure 3:**
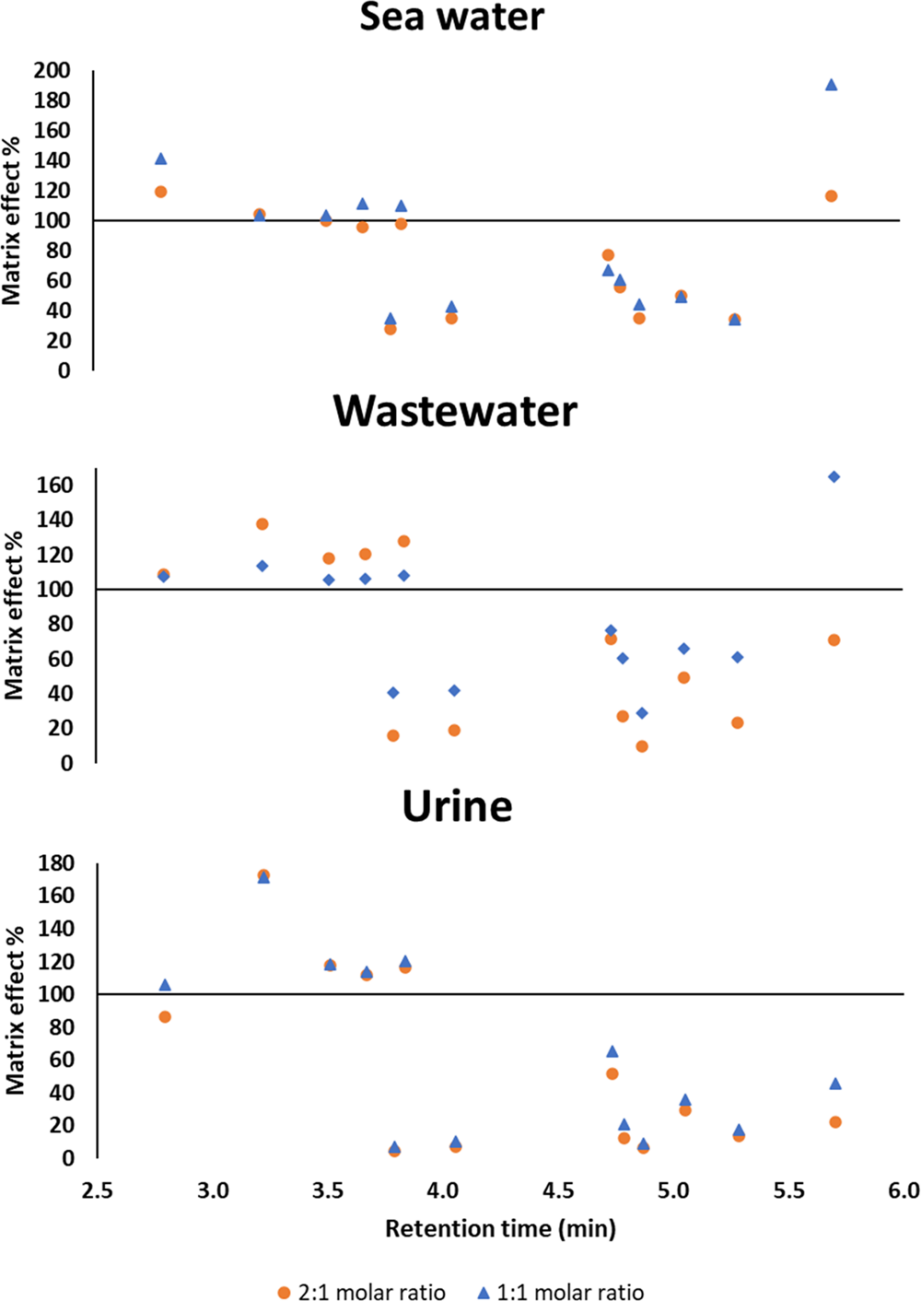
Distribution of the ME (%) vs the retention time (min) of each emerging contaminant for sea water, wastewater, and urine matrix after the application of the DLLME-SFO-UHPLC-MS/MS method.

In addition, this type of calibration allowed the study the ME, whose values are also shown in Table S10 of the Supplementary Material. ME values were calculated using the following equation:^29^ ME (%) = *B*/*A* × 100, where *A* represents the average peak areas of the standard solution in solvent and *B* corresponds to the average peak area of a post-extraction spiked sample. The values obtained can also be seen in Figure 3, where the ME of each analyte in each matrix and with each eutectic mixture is represented against the retention time of each of them. When the ME (%) value ranges between 80 and 120%, no ME is observed. However, ion suppression is considered relevant when ME (%) <80% as well as an important signal enhancement when ME (%) >120%. For all the analytes, similar ME values were observed for both eutectic mixtures, except for DHP in wastewater, which suffers ion suppression when the 2:1 mixture is used, while with the 1:1 molar ratio signal enhancement occurs. As it can be seen in the table, most of the target analytes are dominated by ion suppression, being necessary the development of matrix-matched calibration for the three matrices.

### Analysis of Different Samples

Finally, the developed DLLME-SFO procedure was applied to the analysis of real samples using the eutectic mixture at 1:1 molar ratio due to the similar performance that both solvents showed in the previous studies. Six wastewater samples collected at different WWTPs located on the island of Tenerife (Canary Islands, Spain), two sea water samples collected in two of the beaches located on the island of La Palma (Canary Islands), and one urine sample were analyzed. All of them, as previously described, were filtered through filters with a pore size of 0.22 μm before analysis. Among all the samples, BPA could be detected in wastewater 1 as well as gemfibrozil in wastewaters 1 and 6. However, only gemfibrozil was found at concentrations above the LOQ of the method (0.94 ± 0.88 and 0.92 ± 0.88 μg/L, respectively), being gemfibrozil below the quantifiable concentration in the rest of the wastewater samples.

### NADES Components Toxicity

As previously mentioned, eutectic mixtures that have green properties are preferable, which can be achieved when using natural compounds. Table S11 of the Supplementary Material shows the hazard classification, the risk and safety statements as well as the environmental toxicity of fenchol and acetic acid separately. As can be seen in the table, and judging from available data, it should be noted that both components present a low risk of manipulation and are biodegradable in the environment, which makes them of great interest for the synthesis of environmentally friendly extraction solvents. However, the toxicity of the mixture as a whole must be assessed, even though the toxicity and safety of each of its components also provide important information.

### Greenness Assessment of the Developed Method

The greenness of the method was evaluated using a metric proposed in 2022 by Wojnowski and co-workers^14^ called AGREEprep, which focuses mainly on the sample preparation stage, necessary in the vast majority of analytical procedures. This analytical greenness metric for sample preparation tool is based on the 10 principles of green sample preparation (GSP) and evaluates them quantitatively, giving them a score between 0 (worst performance) and 1 (best performance), and qualitatively using a red-yellow-green scale. It is represented by a pictogram where the center circle indicates the global score of the greenness evaluation of the analytical method and each of the outer sectors corresponds to the 10 principles of the GSP. In addition, a different weight has been assigned to each criterion depending on the impact it has on the greenness of the extraction methodology, which is represented by the sizes of the respective sectors. In this case, the default weights provided by Wojnowski et al.^14^ were considered.

**Figure 4:**
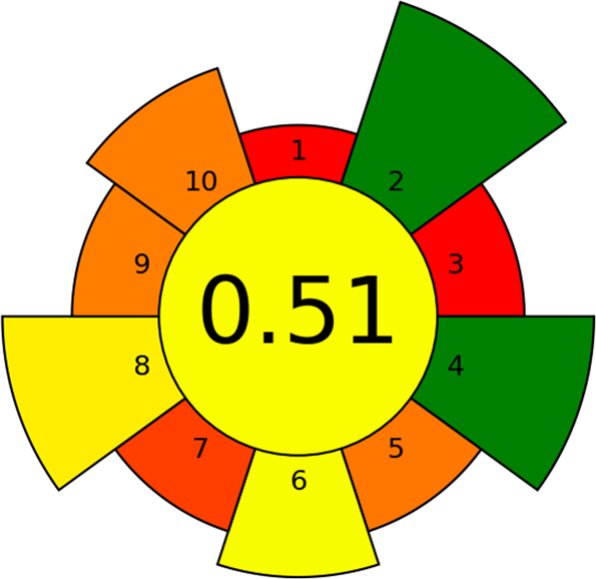
The result of AGREEprep assessment of DLLME-SFO-UHPLC-MS/MS procedure for emerging contaminants determination.

Figure 4 shows the AGREEprep diagram obtained for the sample preparation methodology proposed in this work. This diagram and score were obtained by considering that the DLLME-SFO procedure followed in this work was performed ex situ and no hazardous substances were used. The eutectic mixture synthesized is considered a sustainable solvent and it does not contaminate the sample, so the sample has not been considered as a waste. Regarding other wastes, pipette tips and Pasteur pipettes were not considered as wastes since their use was negligible. The sample throughput was considered to be 9 samples/hour. Regarding the procedure, it was manual, involved four main steps, and it was estimated that ∼80 Wh of power were consumed per sample. In the proposed method, LC-MS/MS was used for the separation and determination of the analytes, and fenchol, acetic acid and ACN were labeled with three distinct hazards symbols (health hazard, flammable and corrosive). Considering these aspects of each of the GSP principles, the final score for the developed DLLME-SFO procedure was 0.51.

### Comparison with Previous Studies

Despite, to the best of our knowledge, the same group of analytes selected in this work has not been previously determined simultaneously in these matrices, but some works can be found in the literature in which DLLME have been used. Several of these works are shown in Table S12 of the Supplementary Material and, as can be seen, a wide variety of extractant solvents has been used, such as alcohols (i.e., dodecanol), chlorinated solvents (i.e., chloroform), ILs or even DESs, although none of them based on fenchol. It is important to highlight that, in most cases, and especially when a DES is not used as extractant, a dispersing solvent is commonly added to obtain a cloud solution, sometimes in volumes of the order of mL, which is not the case of the approach proposed in this work. In general, the performance of the methods is very similar to that of our work, providing good recovery values and LOQs in the μg/L order. However, the fact of using an extraction solvent based on natural compounds, as well as the no need to add a dispersing agent, make the proposed methodology a more sustainable and environmentally friendly approach.

## Conclusions

In this work, the use of fenchol-based eutectic mixtures have been proposed for the first time for the extraction of a group of emerging contaminants from water (sea and waste water) and urine samples. The study of different eutectic mixtures based on the combination of fenchol and acetic acid at different molar ratios (4:1 to 1:4) has provided clear and useful information about their applicability in the DLLME-SFO proposed in this work. Thus, from an operational point of view, only the mixtures 2:1, 1:1, and 1:2 could be applied. However, the latter provided higher extraction unrepeatability, so it was also discarded. Both fenchol–acetic acid mixtures in 2:1 and 1:1 molar ratios showed similar excellent extraction performance, which combined with the fact that a NADES has been used, as well as the simplicity and speed of the procedure, make this methodology a very interesting and robust alternative to ex situ sample preparation methods, as can be seen from the score obtained in the AGREEprep metric. Besides, and concerning the methods applicability to real sample analysis, it can be easily and effectively applied to the determination of these contaminants in both sea and waste water and urine samples, being possible to extend its application to other liquid matrices.

## Supporting Information Available

Chemical structure and properties of the studied emerging contaminants. Operational MS/MS conditions and *m*/*z* transitions of the target compounds. pH and conductivity data of the urine and water samples analyzed in this work. Results of HPLC-UV and UHPLC-MS/MS intraday and interday precision study for the peak areas and the retention times. HPLC-UV and UHPLC-MS/MS external instrumental calibration data of the target analytes. UHPLC-MS/MS external matrix-matched calibration data of the selected emerging contaminants in human urine, wastewater and sea water. Toxicological data of the individual components of the eutectic mixtures used for the extraction of the target analytes. Comparison of previous studies in which common analytes with this work have been extracted using DLLME procedures. HPLC-UV chromatogram of a working solution of the analytes. UHPLC-MS/MS dynamic MRM chromatogram of a wastewater sample spiked with the target analytes. ATR-FTIR spectra of fenchol, acetic acid and the eutectic mixtures. DSC curves obtained for all the eutectic mixtures. Effect of the sample pH on the absolute recovery values of the selected emerging contaminants using fechol:acetic acid at 1:2 molar ratio as DLLME-SFO solvents. Effect of the eutectic mixture volume and extraction temperature during the extraction step on the peak areas of the selected emerging contaminants using 2:1 and 1:1 molar ratio mixtures of fenchol:acetic acid as DLLME-SFO solvents (PDF)

## Abbreviations

4-NP: 4-nonylphenol
4-OP: 4-octylphenol
4-tOP: 4-tert-octylphenol
ACN: acetonitrile
ATR: attenuated total reflectance
BBP: butyl benzyl phthalate
BPA: bisphenol A
BPF: bisphenol F
DBP: dibutyl phthalate
DES: deep eutectic solvent
DHP: dihexyl phthalate
DLLME: dispersive liquid–liquid microextraction
DSC: differential scanning calorimetry
ESI: electrospray ionization
FTIR: Fourier Transform infrared
GSP: green sample preparation
HBA: hydrogen bond acceptor
HBD: hydrogen bond donor
HPLC: high-performance liquid chromatography
LC: liquid chromatography
LOQ: limit of quantification
LPME: liquid-phase microextraction
ME: matrix effect
MRM: multiple reaction monitoring
MS/MS: tandem mass spectrometry
MS: mass spectrometry
NADES: natural deep eutectic solvent
PAE: phthalic acid ester
*R*^2^: determination coefficient
RSD: relative standard deviation
SFO: solidification of the floating organic droplet
UHPLC: ultra-high-performance liquid chromatography
UV: ultraviolet
WWTP: wastewater treatment plant
