# Supplementary material for: Extraction of Emerging Contaminants from Environmental Waters and Urine by Dispersive Liquid–Liquid Microextraction with Solidification of the Floating Organic Droplet Using Fenchol:Acetic Acid Deep Eutectic Mixtures
Source: ACS Sustain Chem Eng. 2022 Nov 16;10(48):15714–25. doi: 10.1021/acssuschemeng.2c04044 (PMC9727775; doi:10.1021/acssuschemeng.2c04044)
Supplement: Supplementary file 1 — sc2c04044_si_001.pdf [file sc2c04044_si_001.pdf]

Extraction of Emerging Contaminants from Environmental Waters and Urine by Dispersive Liquid-Liquid Microextraction with Solidification of the Floating Organic Droplet using Fenchol:Acetic Acid Deep Eutectic Mixtures

Cecilia Ortega-Zamora[†,‡], *Gabriel Jiménez-Skrzypek*[†,‡], *Javier González-Sálamo*[†,‡,⸷**], *Lucia Mazzapioda*[⸷], *Maria Assunta Navarra*[⸷], *Alessandra Gentili*[⸷], *and Javier Hernández-Borges*[†,‡*]

[†]*Departamento de Química, Unidad Departamental de Química Analítica, Facultad de Ciencias, Universidad de La Laguna (ULL). Avda. Astrofísico Fco. Sánchez, s/n. 38206 San Cristóbal de La Laguna, Spain.*

[‡]*Instituto Universitario de Enfermedades Tropicales y Salud Pública de Canarias, Universidad de La Laguna (ULL). Avda. Astrofísico Fco. Sánchez, s/n. 38206 San Cristóbal de La Laguna, Spain.*

[⸷]*Department of Chemistry, Sapienza University of Rome, P.le Aldo Moro, 5. 00185 Rome, Italy.*

***Corresponding author:** Dr. Javier Hernández-Borges

Email: jhborges@ull.edu.es

****Co-corresponding author:** Dr. Javier González-Sálamo

Email: jgsalamo@ull.edu.es

Number of pages: 25; **Number of tables:** 12; **Number of figures:** 7

Table S1. Chemical structure and properties of the studied emerging contaminants.

Analyte, stock solution concentration and solvent	Structure	Molecular formula	MM (g/mol)	Solubility in water (mg/L, 25 ºC)	Vapor pressure (mmHg, 25 ºC)	Log K_{OW}	pKa	Melting point (ºC)	Boiling point (ºC)
BPF (1060 mg/L and MeOH)	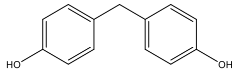	$C_{13}H_{12}O_2$	200.23	Insoluble	$3.70 \cdot 10^{-7}$	2.91	7.55 and 10.80	162.5	390
BPA (970 mg/L and MeOH)	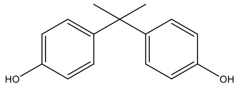	$C_{15}H_{16}O_2$	228.29	120-300[a]	$4.00 \cdot 10^{-8}$	3.32	9.60	153	360.5
17β-estradiol (951 mg/L and MeOH)	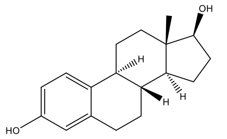	$C_{18}H_{24}O_2$	272.40	3.90[b]	$6.38 \cdot 10^{-9}$	4.01	10.46	178.5	445.9
Testosterone (1035 mg/L and MeOH)	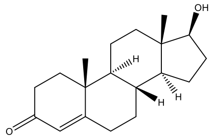	$C_{19}H_{28}O_2$	288.40	23.4	$1.71 \cdot 10^{-8}$	3.32	19.09	155	432.9
Estrone (995 mg/L and MeOH)	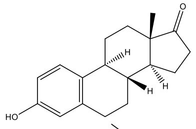	$C_{18}H_{22}O_2$	270.40	30	$2.49 \cdot 10^{-10}$	3.13	10.33	260.2	445.2
Levonorgestrel (1019 mg/L and MeOH)	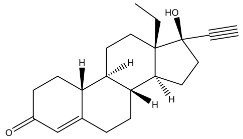	$C_{21}H_{28}O_2$	312.40	2.05	$3.92 \cdot 10^{-10}$	3.48	17.91	240	459.1
Gemfibrozil (1005 mg/L and MeOH)	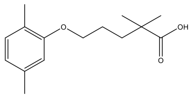	$C_{15}H_{22}O_3$	250.33	11	$3.10 \cdot 10^{-5}$	4.77	4.50	62	159
4-tOP (989 mg/L and MeOH)	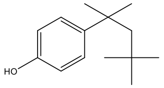	$C_{14}H_{22}O$	206.32	19.00[c]	$4.78 \cdot 10^{-4}$	5.25	10.15	84.5	279
BBP (1024 mg/L and ACN)	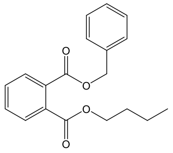	$C_{19}H_{20}O_4$	312.40	2.69	$8.25 \cdot 10^{-6}$	4.73	-	-35	370

DBP (905 mg/L and ACN)	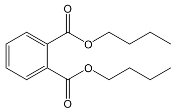	$C_{16}H_{22}O_4$	278.34	11.2	$2.01·10^{-5}$	4.57	-	-35	340
4-OP (1109 mg/L and MeOH)	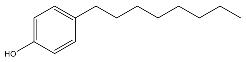	$C_{14}H_{22}O$	206.32	12.60[c]	$2.50·10^{-4}$	5.28	10.15	44-45	280
4-NP (1069 mg/L and MeOH)	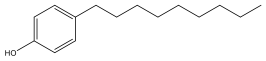	$C_{15}H_{24}O$	220.35	4.90[c]	$8.18·10^{-4}$	5.76	10.31	42	317
DHP (952 mg/L and ACN)	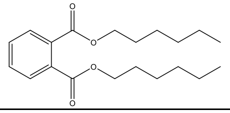	$C_{20}H_{30}O_4$	334.40	0.05	$1.40·10^{-5}$	6.82	-	-58	210

[a)]Solubility increases with pH at constant temperature, being the most common value 300 ºC, [b)]27 ºC, [c)]From reference Salgueiro-González *et al*. [Salgueiro-González, N.; Muniategui-Lorenzo, S.; López-Mahía, P.; Prada-Rodríguez, D. Trends in Analytical Methodologies for the Determination of Alkylphenols and Bisphenol A in Water Samples. *Anal. Chim. Acta* **2017**, *962*, 1-14. https://doi.org/10.1016/j.aca.2017.01.035]. Data taken from SciFinder® and PubChem databases. MM: Molecular mass.

Table S2. Operational MS/MS conditions and the *m/z* transitions of the target compounds.

Analyte	Retention time (min)[a]	Precursor ion (*m/z*)	Product ion (*m/z*)		Fragmentor (V)		CE (eV)		Cell accelerator (V)	Polarity
			Quantifier	Qualifier	Quantifier	Qualifier	Quantifier	Qualifier		
Gemfibrozil	2.80 ± 0.01	249.1	121.0	127.0	100	90	13	10	4	Negative
BPF	3.21 ± 0.01	199.1	93.1	105.1	140	140	22	25	4	Negative
BPA	3.51 ± 0.01	227.0	212.0	211.1	130	130	20	35	4	Negative
17β-estradiol	3.67 ± 0.01	271.2	145.1	183.1	150	150	45	45	4	Negative
Testosterone	3.80 ± 0.01	289.2	97.1	109.1	150	150	20	20	4	Positive
Estrone	3.84 ± 0.01	269.2	145.1	159.1	150	150	40	46	4	Negative
Levonorgestrel	4.06 ± 0.01	313.2	245.2	109.1	150	150	20	40	4	Positive
4-tOP	4.74 ± 0.01	205.0	205.0	133.0	150	150	0	33	4	Negative
BBP	4.79 ± 0.01	313.0	91.0	205.0	72	80	5	45	3	Positive
DBP	4.88 ± 0.01	279.0	149.0	121.0	72	72	15	45	3	Positive
4-OP	5.06 ± 0.01	205.0	205.0	106.0	160	160	0	30	4	Negative
4-NP	5.29 ± 0.01	219.1	219.1	106.1	130	130	0	22	4	Negative
DHP	5.70 ± 0.01	335.0	149.0	233.0	80	72	5	18	3	Positive

CE: collision energy. [a]Standard deviation of 40 injections.

Table S3. pH and conductivity data of the urine and water samples analyzed in this work.

	pH	Conductivity at 25 ºC
	**Urine**	
Sample 1	4.84	25.60 mS/cm
	**Wastewater**	
Sample 1	8.01	2040 µS/cm
Sample 2	8.96	1448 µS/cm
Sample 3	7.81	1155 µS/cm
Sample 4	8.78	603 µS/cm
Sample 5	9.06	1523 µS/cm
Sample 6	8.82	1918 µS/cm
	**Sea water**	
Sample 1	8.08	59.30 mS/cm
Sample 2	8.18	50.90 mS/cm

Sample 1 of each type were also used for validation purposes.

Table S4. Results of HPLC-UV intraday and interday precision study for the peak areas.

**Analyte**	**Level 1: 100 μg/L**				**Level 2: 500 μg/L**				**Level 3: 1000 μg/L**			
	**% RSD intraday (n=5)**			**% RSD interday (n=15)**	**% RSD intraday (n=5)**			**% RSD interday (n=15)**	**% RSD intraday (n=5)**			**% RSD interday (n=15)**
	**Day 1**	**Day 2**	**Day 3**		**Day 1**	**Day 2**	**Day 3**		**Day 1**	**Day 2**	**Day 3**	
BPF	1.1	1.2	2.6	4.6	0.4	1.6	0.3	3.9	0.4	1.8	0.2	5.5
BPA	0.6	1.7	2.9	4.9	0.3	3.4	0.4	2.9	0.4	1.7	0.1	4.2
17β-estradiol	3.3	2.6	3.5	6.9	0.5	1.6	0.9	5.6	0.9	2.0	0.4	1.5
Testosterone	3.4	2.6	4.2	4.0	0.7	3.5	2.1	3.4	4.0	1.8	0.4	7.2
Estrone	1.8	3.2	4.2	5.5	1.2	2.4	0.6	4.0	1.2	2.4	0.3	1.8
Levonorgestrel	0.7	1.4	2.1	3.9	0.4	1.9	0.3	5.3	0.3	2.6	0.3	4.6
Gemfibrozil	2.3	1.4	3.8	6.2	0.5	2.4	0.4	5.1	0.6	1.7	0.3	2.4
4-tOP	1.0	2.4	2.8	5.7	0.3	2.3	0.4	4.8	0.3	2.5	0.4	5.9
BBP	0.8	1.8	3.6	7.2	0.4	2.6	0.6	4.8	0.2	2.2	0.2	5.5
DBP	3.4	3.3	3.6	6.0	0.3	1.6	1.3	3.4	0.4	2.4	1.2	4.5
4-OP	1.4	2.1	3.2	6.5	0.4	2.7	0.5	2.1	0.5	2.3	0.2	7.1
4-NP	3.4	3.5	2.4	6.6	0.3	2.3	0.4	3.4	0.3	1.9	0.4	1.2
DHP	2.0	2.0	2.1	4.7	0.3	2.0	0.4	3.2	0.4	2.3	0.7	3.5

Table S5. Results of HPLC-UV intraday and interday precision study for the retention times.

Analyte	Level 1: 100 μg/L				Level 2: 500 μg/L				Level 3: 1000 μg/L			
	% RSD intraday (n=5)			% RSD interday (n=15)	% RSD intraday (n=5)			% RSD interday (n=15)	% RSD intraday (n=5)			% RSD interday (n=15)
	Day 1	Day 2	Day 3		Day 1	Day 2	Day 3		Day 1	Day 2	Day 3	
BPF	0.08	0.08	0.05	0.11	0.07	0.11	0.07	0.10	0.09	0.08	0.11	0.12
BPA	0.09	0.09	0.09	0.12	0.05	0.06	0.06	0.12	0.08	0.11	0.08	0.11
17β-estradiol	0.09	0.08	0.08	0.12	0.04	0.08	0.07	0.09	0.03	0.08	0.06	0.06
Testosterone	0.11	0.09	0.11	0.11	0.02	0.07	0.09	0.13	0.08	0.10	0.04	0.09
Estrone	0.10	0.11	0.10	0.12	0.06	0.06	0.07	0.08	0.06	0.09	0.07	0.10
Levonorgestrel	0.08	0.08	0.09	0.12	0.05	0.03	0.07	0.11	0.03	0.11	0.04	0.12
Gemfibrozil	0.06	0.07	0.06	0.08	0.04	0.03	0.04	0.04	0.08	0.06	0.04	0.11
4-tOP	0.06	0.05	0.07	0.08	0.02	0.04	0.04	0.10	0.09	0.07	0.03	0.10
BBP	0.06	0.06	0.06	0.08	0.04	0.02	0.04	0.05	0.10	0.06	0.02	0.10
DBP	0.04	0.06	0.06	0.07	0.02	0.03	0.03	0.05	0.10	0.07	0.03	0.10
4-OP	0.04	0.05	0.05	0.06	0.02	0.03	0.03	0.04	0.08	0.06	0.04	0.09
4-NP	0.05	0.05	0.06	0.06	0.02	0.02	0.03	0.03	0.06	0.05	0.03	0.07
DHP	0.04	0.04	0.05	0.05	0.02	0.04	0.04	0.08	0.04	0.04	0.04	0.05

Table S6. Results of UHPLC-MS/MS intraday and interday precision study for the peak areas.

Analyte	Level 1: 10 µg/L				Level 2: 50 µg/L				Level 3: 100 µg/L			
	% RSD intraday (n=5)			% RSD interday (n=15)	% RSD intraday (n=5)			% RSD interday (n=15)	% RSD intraday (n=5)			% RSD interday (n=15)
	Day 1	Day 2	Day 3		Day 1	Day 2	Day 3		Day 1	Day 2	Day 3	
BPF	3.9	11.5	7.9	13.9	6.4	7.3	4.7	15.3	6.2	3.0	3.7	11.0
BPA	3.6	1.3	3.8	15.3	2.6	2.3	2.5	9.8	1.2	1.3	4.1	6.7
17β-estradiol	6.7	6.1	2.8	10.7	3.7	1.0	4.2	9.5	2.9	2.4	1.9	7.1
Testosterone	0.8	0.9	0.4	17.8	0.6	2.8	1.3	14.5	2.2	2.0	3.2	11.8
Estrone	4.5	2.8	2.5	8.4	0.8	1.0	1.7	4.2	2.0	1.4	1.5	6.7
Levonorgestrel	2.1	0.9	1.1	15.6	0.4	1.9	2.0	12.6	2.0	1.2	2.3	10.4
Gemfibrozil	1.0	3.1	1.2	8.4	1.2	1.0	0.9	7.1	2.0	1.0	5.2	7.7
4-tOP	2.9	5.0	5.2	5.1	1.3	2.3	1.7	5.5	1.9	1.2	1.3	7.5
BBP	1.0	1.4	1.2	8.1	0.7	2.2	0.7	6.7	10.5	1.1	2.1	7.2
DBP	0.5	3.9	1.0	17.4	0.9	0.9	0.6	12.3	4.8	0.9	1.4	8.8
4-OP	3.6	3.7	5.6	6.7	3.0	2.2	1.8	6.9	1.6	2.7	1.2	7.6
4-NP	0.8	3.2	2.6	7.1	1.8	3.3	2.3	6.9	2.6	1.6	1.9	7.7
DHP	1.3	1.2	1.6	8.9	1.6	1.6	1.3	5.8	9.7	1.3	1.9	6.3

Table S7. Results of UHPLC-MS/MS intraday and interday precision study for the retention times.

Analyte	Level 1: 10 μg/L				Level 2: 50 μg/L				Level 3: 100 μg/L			
	% RSD intraday (n=5)			% RSD interday (n=15)	% RSD intraday (n=5)			% RSD interday (n=15)	% RSD intraday (n=5)			% RSD interday (n=15)
	Day 1	Day 2	Day 3		Day 1	Day 2	Day 3		Day 1	Day 2	Day 3	
BPF	0.12	0.14	0.12	0.16	0.00	0.00	0.12	0.13	0.00	0.12	0.00	0.15
BPA	0.00	0.11	0.00	0.12	0.00	0.00	0.00	0.12	0.11	0.00	0.00	0.11
17β-estradiol	0.00	0.10	0.00	0.18	0.10	0.10	0.00	0.14	0.12	0.00	0.12	0.11
Testosterone	0.10	0.00	0.00	0.19	0.00	0.10	0.00	0.14	0.00	0.00	0.12	0.11
Estrone	0.10	0.10	0.10	0.21	0.12	0.00	0.00	0.11	0.10	0.00	0.00	0.11
Levonorgestrel	0.00	0.11	0.00	0.22	0.00	0.00	0.00	0.17	0.00	0.09	0.09	0.16
Gemfibrozil	0.00	0.12	0.00	0.20	0.00	0.00	0.00	0.15	0.00	0.13	0.00	0.14
4-tOP	0.00	0.00	0.00	0.23	0.00	0.00	0.00	0.23	0.00	0.08	0.00	0.19
BBP	0.08	0.00	0.08	0.24	0.08	0.08	0.00	0.21	0.00	0.00	0.00	0.15
DBP	0.00	0.08	0.00	0.24	0.08	0.09	0.00	0.19	0.00	0.00	0.00	0.14
4-OP	0.00	0.00	0.00	0.29	0.00	0.00	0.00	0.29	0.00	0.00	0.00	0.24
4-NP	0.00	0.00	0.00	0.28	0.00	0.00	0.00	0.28	0.00	0.07	0.00	0.28
DHP	0.07	0.07	0.07	0.36	0.08	0.00	0.08	0.36	0.00	0.08	0.08	0.32

Table S8. HPLC-UV external instrumental calibration data of the target analytes.

Analyte	Studied linear range (μg/L)	Regression equation (n=8)		$s_{y/x}$	R^2
		$b \pm s_b \cdot t_{(0.05;6)}$	$a \pm s_a \cdot t_{(0.05;6)}$		
BPF	25-1000	$3.44 \cdot 10^5 \pm 3.85 \cdot 10^3$	$-2.51 \cdot 10^3 \pm 1.87 \cdot 10^3$	$1.52 \cdot 10^3$	0.9999
BPA	25-1000	$2.90 \cdot 10^5 \pm 3.21 \cdot 10^3$	$-1.87 \cdot 10^3 \pm 1.56 \cdot 10^3$	$1.27 \cdot 10^3$	0.9999
17β-estradiol	38-1500	$6.94 \cdot 10^4 \pm 9.52 \cdot 10^2$	$-1.05 \cdot 10^3 \pm 6.96 \cdot 10^2$	$5.65 \cdot 10^2$	0.9998
Testosterone	25-1000	$2.46 \cdot 10^5 \pm 3.26 \cdot 10^3$	$-2.28 \cdot 10^3 \pm 1.59 \cdot 10^3$	$1.29 \cdot 10^3$	0.9998
Estrone	38-1500	$6.85 \cdot 10^4 \pm 1.64 \cdot 10^3$	$-1.71 \cdot 10^3 \pm 1.20 \cdot 10^3$	$9.72 \cdot 10^2$	0.9994
Levonorgestrel	25-1000	$2.49 \cdot 10^5 \pm 3.48 \cdot 10^3$	$-2.58 \cdot 10^3 \pm 1.70 \cdot 10^3$	$1.38 \cdot 10^3$	0.9998
Gemfibrozil	25-1000	$7.05 \cdot 10^4 \pm 2.83 \cdot 10^3$	$-1.60 \cdot 10^3 \pm 1.38 \cdot 10^3$	$1.12 \cdot 10^3$	0.9984
4-tOP	25-1000	$1.81 \cdot 10^5 \pm 2.71 \cdot 10^3$	$-1.76 \cdot 10^3 \pm 1.32 \cdot 10^3$	$1.07 \cdot 10^3$	0.9998
BBP	25-1000	$1.46 \cdot 10^5 \pm 2.06 \cdot 10^3$	$-1.47 \cdot 10^3 \pm 1.01 \cdot 10^3$	$8.17 \cdot 10^2$	0.9998
DBP	25-1000	$1.43 \cdot 10^5 \pm 2.81 \cdot 10^3$	$-2.19 \cdot 10^3 \pm 1.37 \cdot 10^3$	$1.11 \cdot 10^3$	0.9996
4-OP	25-1000	$1.50 \cdot 10^5 \pm 2.78 \cdot 10^3$	$-2.09 \cdot 10^3 \pm 1.35 \cdot 10^3$	$1.10 \cdot 10^3$	0.9997
4-NP	25-1000	$1.49 \cdot 10^5 \pm 2.93 \cdot 10^3$	$-2.33 \cdot 10^3 \pm 1.43 \cdot 10^3$	$1.16 \cdot 10^3$	0.9996
DHP	25-1000	$1.26 \cdot 10^5 \pm 3.28 \cdot 10^3$	$-1.72 \cdot 10^3 \pm 1.60 \cdot 10^3$	$1.30 \cdot 10^3$	0.9993

b: slope; s_b: standard deviation of the slope; a: intercept; s_a: standard deviation of the intercept; $s_{y/x}$: standard deviation of the estimate; R^2: determination coefficient.

Table S9. UHPLC-MS/MS external instrumental calibration data of the target analytes.

Analyte	Studied linear range (μg/L)	Regression equation (n=8)		$s_{y/x}$	R^2
		$b \pm s_b \cdot t_{(0.05;6)}$	$a \pm s_a \cdot t_{(0.05;6)}$		
BPF	1-150	$9.25 \cdot 10 \pm 7.85$	$-4.53 \cdot 10^2 \pm 5.68 \cdot 10^2$	$4.44 \cdot 10^2$	0.9928
BPA	1-150	$7.66 \cdot 10^2 \pm 3.73 \cdot 10$	$-1.11 \cdot 10^3 \pm 2.70 \cdot 10^3$	$2.11 \cdot 10^3$	0.9976
17β-estradiol	1.5-225	$2.73 \cdot 10^2 \pm 1.54 \cdot 10$	$-1.07 \cdot 10^3 \pm 1.67 \cdot 10^3$	$1.30 \cdot 10^3$	0.9968
Testosterone	1-150	$1.24 \cdot 10^5 \pm 4.61 \cdot 10^3$	$2.74 \cdot 10^5 \pm 3.33 \cdot 10^5$	$2.60 \cdot 10^5$	0.9986
Estrone	1.5-225	$1.10 \cdot 10^3 \pm 5.06 \cdot 10$	$-3.88 \cdot 10^3 \pm 5.48 \cdot 10^3$	$4.29 \cdot 10^3$	0.9979
Levonorgestrel	1-150	$2.52 \cdot 10^4 \pm 1.03 \cdot 10^3$	$5.18 \cdot 10^4 \pm 7.41 \cdot 10^4$	$5.79 \cdot 10^4$	0.9983
Gemfibrozil	1-150	$1.88 \cdot 10^3 \pm 6.30 \cdot 10$	$-3.17 \cdot 10^3 \pm 4.55 \cdot 10^3$	$3.56 \cdot 10^3$	0.9989
4-tOP	1-150	$5.87 \cdot 10^3 \pm 2.38 \cdot 10^2$	$-1.59 \cdot 10^4 \pm 1.72 \cdot 10^4$	$1.35 \cdot 10^4$	0.9983
BBP	1-150	$2.70 \cdot 10^5 \pm 1.76 \cdot 10^4$	$5.96 \cdot 10^5 \pm 1.27 \cdot 10^6$	$9.95 \cdot 10^5$	0.9958
DBP	1-150	$3.00 \cdot 10^5 \pm 2.80 \cdot 10^4$	$4.56 \cdot 10^7 \pm 2.03 \cdot 10^6$	$1.58 \cdot 10^6$	0.9913
4-OP	1-150	$5.71 \cdot 10^3 \pm 1.77 \cdot 10^2$	$-1.07 \cdot 10^4 \pm 1.28 \cdot 10^4$	$9.98 \cdot 10^3$	0.9990
4-NP	1-150	$6.67 \cdot 10^3 \pm 1.47 \cdot 10^2$	$-9.51 \cdot 10^3 \pm 1.06 \cdot 10^4$	$8.31 \cdot 10^3$	0.9995
DHP	1-150	$8.93 \cdot 10^4 \pm 2.36 \cdot 10^3$	$1.34 \cdot 10^5 \pm 1.71 \cdot 10^5$	$1.33 \cdot 10^5$	0.9993

b: slope; s_b: standard deviation of the slope; a: intercept; s_a: standard deviation of the intercept; $s_{y/x}$: standard deviation of the estimate; R^2: determination coefficient.

Table S10. UHPLC-MS/MS external matrix-matched calibration data of the selected emerging contaminants in human urine, wastewater and sea water.

Analyte	Molar ratio eutectic mixture (fenchol:acetic acid)	Sample	Studied linear range (μg/L)	Regression equation (n=8)		$s_{y/x}$	R^2	ME %	LOQ_{method} (μg/L)
				$b \pm s_b \cdot t_{(0.05;6)}$	$a \pm s_a \cdot t_{(0.05;6)}$				
BPF	2:1	Urine	1-150	$1.29{\cdot}10^2 \pm 5.75$	$1.33{\cdot}10^2 \pm 4.16{\cdot}10^2$	$3.25{\cdot}10^2$	0.9980	173	0.15
		Wastewater	1-150	$9.93{\cdot}10 \pm 4.88$	$1.98{\cdot}10^2 \pm 3.53{\cdot}10^2$	$2.76{\cdot}10^2$	0.9976	138	0.21
		Sea water	1-150	$7.34{\cdot}10 \pm 4.98$	$1.08{\cdot}10^2 \pm 3.60{\cdot}10^2$	$2.81{\cdot}10^2$	0.9954	105	0.20
	1:1	Urine	1-150	$1.24{\cdot}10^2 \pm 4.97$	$2.94{\cdot}10^2 \pm 3.59{\cdot}10^2$	$2.81{\cdot}10^2$	0.9984	171	0.21
		Wastewater	1-150	$9.31{\cdot}10 \pm 4.11$	$1.04{\cdot}10^2 \pm 2.97{\cdot}10^2$	$2.32{\cdot}10^2$	0.9981	114	0.19
		Sea water	1-150	$7.28{\cdot}10 \pm 5.58$	$1.85{\cdot}10^2 \pm 4.03{\cdot}10^2$	$3.15{\cdot}10^2$	0.9941	104	0.22
BPA	2:1	Urine	1-150	$8.53{\cdot}10^2 \pm 2.17{\cdot}10$	$-3.87{\cdot}10^2 \pm 1.57{\cdot}10^3$	$1.23{\cdot}10^3$	0.9994	118	0.13
		Wastewater	1-150	$8.50{\cdot}10^2 \pm 3.04{\cdot}10$	$1.29{\cdot}10^3 \pm 2.20{\cdot}10^3$	$1.72{\cdot}10^3$	0.9987	118	0.14
		Sea water	1-150	$6.38{\cdot}10^2 \pm 5.36{\cdot}10$	$1.40{\cdot}10^3 \pm 3.88{\cdot}10^3$	$3.03{\cdot}10^3$	0.9930	100	0.17
	1:1	Urine	1-150	$8.24{\cdot}10^2 \pm 3.04{\cdot}10$	$1.14{\cdot}10^3 \pm 2.20{\cdot}10^3$	$1.72{\cdot}10^3$	0.9986	119	0.14
		Wastewater	1-150	$7.69{\cdot}10^2 \pm 1.12{\cdot}10$	$-6.10{\cdot}10^2 \pm 8.12{\cdot}10^2$	$6.35{\cdot}10^2$	0.9998	106	0.13
		Sea water	1-150	$6.61{\cdot}10^2 \pm 5.11{\cdot}10$	$1.69{\cdot}10^3 \pm 3.69{\cdot}10^3$	$2.89{\cdot}10^3$	0.9940	104	0.24
17β-estradiol	2:1	Urine	1.5-225	$2.83{\cdot}10^2 \pm 6.32$	$3.60{\cdot}10 \pm 6.85{\cdot}10^2$	$5.36{\cdot}10^2$	0.9995	112	0.26
		Wastewater	1.5-225	$3.05{\cdot}10^2 \pm 9.82$	$2.89{\cdot}10^2 \pm 1.06{\cdot}10^3$	$8.32{\cdot}10^2$	0.9990	121	0.27
		Sea water	1.5-225	$2.32{\cdot}10^2 \pm 1.83{\cdot}10$	$1.32{\cdot}10^3 \pm 1.99{\cdot}10^3$	$1.55{\cdot}10^3$	0.9938	96	0.39
	1:1	Urine	1.5-225	$2.76{\cdot}10^2 \pm 1.06{\cdot}10$	$5.54{\cdot}10^2 \pm 1.15{\cdot}10^3$	$8.99{\cdot}10^2$	0.9985	114	0.25
		Wastewater	1.5-225	$2.62{\cdot}10^2 \pm 5.77$	$1.11{\cdot}10^2 \pm 6.25{\cdot}10^2$	$4.89{\cdot}10^2$	0.9995	106	0.29
		Sea water	1.5-225	$2.79{\cdot}10^2 \pm 1.67{\cdot}10$	$-2.62{\cdot}10^2 \pm 1.81{\cdot}10^3$	$1.41{\cdot}10^3$	0.9964	112	0.25
Testosterone	2:1	Urine	1-150	$5.92{\cdot}10^3 \pm 3.69{\cdot}10^2$	$-1.88{\cdot}10^4 \pm 2.67{\cdot}10^4$	$2.09{\cdot}10^4$	0.9961	5	0.18
		Wastewater	1-150	$2.17{\cdot}10^4 \pm 9.23{\cdot}10^2$	$-3.49{\cdot}10^4 \pm 6.67{\cdot}10^4$	$5.21{\cdot}10^4$	0.9982	16	0.21
		Sea water	1-150	$3.61{\cdot}10^4 \pm 2.74{\cdot}10^3$	$-1.34{\cdot}10^5 \pm 1.98{\cdot}10^5$	$1.55{\cdot}10^5$	0.9943	28	0.26
	1:1	Urine	1-150	$9.32{\cdot}10^3 \pm 6.49{\cdot}10^2$	$1.52{\cdot}10^3 \pm 4.69{\cdot}10^4$	$3.66{\cdot}10^4$	0.9952	7	0.21

		Wastewater	1-150	$5.09\cdot10^4 \pm 1.93\cdot10^3$	$9.92\cdot10^4 \pm 1.39\cdot10^5$	$1.09\cdot10^5$	0.9986	41	0.25
		Sea water	1-150	$4.18\cdot10^4 \pm 3.28\cdot10^3$	$1.88\cdot10^5 \pm 2.37\cdot10^5$	$1.85\cdot10^5$	0.9939	35	0.24
Estrone	2:1	Urine	1.5-225	$1.23\cdot10^3 \pm 3.19\cdot10$	$-1.98\cdot10^3 \pm 3.46\cdot10^3$	$2.71\cdot10^3$	0.9993	117	0.23
		Wastewater	1.5-225	$1.28\cdot10^3 \pm 5.21\cdot10$	$2.01\cdot10^3 \pm 5.65\cdot10^3$	$4.41\cdot10^3$	0.9983	128	0.26
		Sea water	1.5-225	$1.00\cdot10^3 \pm 7.37\cdot10$	$4.01\cdot10^3 \pm 7.99\cdot10^3$	$6.25\cdot10^3$	0.9946	98	0.34
	1:1	Urine	1.5-225	$1.20\cdot10^3 \pm 4.79\cdot10$	$2.26\cdot10^3 \pm 5.19\cdot10^3$	$4.06\cdot10^3$	0.9984	121	0.24
		Wastewater	1.5-225	$1.08\cdot10^3 \pm 2.93\cdot10$	$1.27\cdot10^2 \pm 3.18\cdot10^3$	$2.48\cdot10^3$	0.9993	108	0.26
		Sea water	1.5-225	$1.04\cdot10^3 \pm 5.60\cdot10$	$2.58\cdot10^3 \pm 6.07\cdot10^3$	$4.75\cdot10^3$	0.9971	110	0.19
Levonorgestrel	2:1	Urine	1-150	$1.84\cdot10^3 \pm 1.47\cdot10^2$	$-6.59\cdot10^3 \pm 1.06\cdot10^4$	$8.28\cdot10^3$	0.9937	7	0.18
		Wastewater	1-150	$5.03\cdot10^3 \pm 2.47\cdot10^2$	$-5.85\cdot10^3 \pm 1.79\cdot10^4$	$1.40\cdot10^4$	0.9976	19	0.19
		Sea water	1-150	$9.15\cdot10^3 \pm 4.61\cdot10^2$	$-2.03\cdot10^4 \pm 3.33\cdot10^4$	$2.60\cdot10^4$	0.9975	35	0.26
	1:1	Urine	1-150	$2.82\cdot10^3 \pm 1.81\cdot10^2$	$-9.97\cdot10^2 \pm 1.31\cdot10^4$	$1.02\cdot10^4$	0.9959	10	0.23
		Wastewater	1-150	$1.02\cdot10^4 \pm 7.38\cdot10^2$	$2.66\cdot10^4 \pm 5.33\cdot10^4$	$4.17\cdot10^4$	0.9947	42	0.17
		Sea water	1-150	$1.07\cdot10^4 \pm 8.11\cdot10^2$	$5.12\cdot10^4 \pm 5.86\cdot10^4$	$4.58\cdot10^4$	0.9943	43	0.17
Gemfibrozil	2:1	Urine	1-150	$1.66\cdot10^3 \pm 1.56\cdot10^2$	$8.41\cdot10^2 \pm 1.12\cdot10^4$	$8.79\cdot10^3$	0.9913	86	0.15
		Wastewater	1-150	$1.82\cdot10^3 \pm 8.41\cdot10$	$1.03\cdot10^4 \pm 6.08\cdot10^3$	$4.75\cdot10^3$	0.9979	109	0.16
		Sea water	1-150	$2.08\cdot10^3 \pm 7.70\cdot10$	$-6.84\cdot10^2 \pm 5.57\cdot10^3$	$4.35\cdot10^3$	0.9986	119	0.23
	1:1	Urine	1-150	$1.81\cdot10^3 \pm 1.08\cdot10^2$	$2.76\cdot10^3 \pm 7.79\cdot10^3$	$6.09\cdot10^3$	0.9964	106	0.16
		Wastewater	1-150	$1.85\cdot10^3 \pm 9.07\cdot10$	$-1.67\cdot10^3 \pm 6.55\cdot10^3$	$5.12\cdot10^3$	0.9976	107	0.12
		Sea water	1-150	$2.35\cdot10^3 \pm 1.22\cdot10^2$	$4.42\cdot10^3 \pm 8.78\cdot10^3$	$6.86\cdot10^3$	0.9973	142	0.14
4-tOP	2:1	Urine	1-150	$3.09\cdot10^3 \pm 1.85\cdot10^2$	$-2.02\cdot10^3 \pm 1.33\cdot10^4$	$1.04\cdot10^4$	0.9964	51	0.15
		Wastewater	1-150	$3.84\cdot10^3 \pm 1.53\cdot10^2$	$-4.09\cdot10^3 \pm 1.10\cdot10^4$	$8.63\cdot10^3$	0.9984	72	0.17
		Sea water	1-150	$3.84\cdot10^3 \pm 2.95\cdot10^2$	$4.93\cdot10^3 \pm 2.13\cdot10^4$	$1.67\cdot10^4$	0.9941	77	0.25
	1:1	Urine	1-150	$3.54\cdot10^3 \pm 1.61\cdot10^2$	$1.97\cdot10^3 \pm 1.16\cdot10^4$	$9.09\cdot10^3$	0.9979	66	0.17
		Wastewater	1-150	$4.17\cdot10^3 \pm 1.70\cdot10^2$	$-5.51\cdot10^3 \pm 1.23\cdot10^4$	$9.58\cdot10^3$	0.9983	77	0.17

		Sea water	1-150	$3.91\cdot10^{3} \pm 1.25\cdot10^{2}$	$-3.61\cdot10^{2} \pm 9.02\cdot10^{3}$	$7.05\cdot10^{3}$	0.9990	68	0.17
BBP	2:1	Urine	1-150	$4.01\cdot10^{4} \pm 2.98\cdot10^{3}$	$-3.23\cdot10^{4} \pm 2.15\cdot10^{5}$	$1.68\cdot10^{5}$	0.9945	13	0.17
		Wastewater	1-150	$7.42\cdot10^{4} \pm 4.24\cdot10^{3}$	$5.07\cdot10^{4} \pm 3.06\cdot10^{5}$	$2.39\cdot10^{5}$	0.9967	27	0.20
		Sea water	1-150	$1.58\cdot10^{5} \pm 8.16\cdot10^{3}$	$-1.38\cdot10^{5} \pm 5.90\cdot10^{5}$	$4.61\cdot10^{5}$	0.9973	56	0.18
	1:1	Urine	1-150	$5.75\cdot10^{4} \pm 3.68\cdot10^{3}$	$1.50\cdot10^{5} \pm 2.66\cdot10^{5}$	$2.08\cdot10^{5}$	0.9959	21	0.15
		Wastewater	1-150	$1.64\cdot10^{5} \pm 1.04\cdot10^{4}$	$6.16\cdot10^{5} \pm 7.51\cdot10^{5}$	$5.87\cdot10^{5}$	0.9960	61	0.20
		Sea water	1-150	$1.78\cdot10^{5} \pm 1.34\cdot10^{4}$	$7.08\cdot10^{5} \pm 9.67\cdot10^{5}$	$7.56\cdot10^{5}$	0.9944	61	0.18
DBP	2:1	Urine	1-150	$5.99\cdot10^{4} \pm 5.02\cdot10^{3}$	$4.30\cdot10^{5} \pm 3.63\cdot10^{5}$	$2.83\cdot10^{5}$	0.9930	7	0.16
		Wastewater	1-150	$8.64\cdot10^{4} \pm 4.67\cdot10^{3}$	$2.00\cdot10^{5} \pm 3.37\cdot10^{5}$	$2.64\cdot10^{5}$	0.9971	10	0.17
		Sea water	1-150	$2.34\cdot10^{5} \pm 2.13\cdot10^{4}$	$4.44\cdot10^{6} \pm 1.54\cdot10^{6}$	$1.20\cdot10^{6}$	0.9918	35	0.25
	1:1	Urine	1-150	$7.23\cdot10^{4} \pm 3.68\cdot10^{3}$	$1.50\cdot10^{5} \pm 2.66\cdot10^{5}$	$2.08\cdot10^{5}$	0.9959	9	0.16
		Wastewater	1-150	$1.55\cdot10^{5} \pm 1.16\cdot10^{4}$	$7.50\cdot10^{6} \pm 8.35\cdot10^{5}$	$6.53\cdot10^{5}$	0.9945	29	0.18
		Sea water	1-150	$1.99\cdot10^{5} \pm 1.55\cdot10^{4}$	$1.47\cdot10^{7} \pm 1.12\cdot10^{6}$	$8.75\cdot10^{5}$	0.9940	45	0.16
4-OP	2:1	Urine	5-150	$1.85\cdot10^{3} \pm 1.83\cdot10^{2}$	$-5.43\cdot10^{3} \pm 1.41\cdot10^{4}$	$9.00\cdot10^{3}$	0.9927	30	0.16
		Wastewater	5-150	$2.63\cdot10^{3} \pm 1.61\cdot10^{2}$	$-6.02\cdot10^{3} \pm 1.24\cdot10^{4}$	$7.89\cdot10^{3}$	0.9972	49	0.17
		Sea water	1-150	$2.55\cdot10^{3} \pm 1.98\cdot10^{2}$	$5.64\cdot10^{3} \pm 1.43\cdot10^{4}$	$1.12\cdot10^{4}$	0.9940	50	0.26
	1:1	Urine	5-150	$2.08\cdot10^{3} \pm 2.66\cdot10$	$-7.32\cdot10^{3} \pm 2.05\cdot10^{3}$	$1.31\cdot10^{3}$	0.9999	36	0.17
		Wastewater	1-150	$3.69\cdot10^{3} \pm 1.27\cdot10^{2}$	$-7.87\cdot10^{3} \pm 9.18\cdot10^{3}$	$7.17\cdot10^{3}$	0.9988	66	0.18
		Sea water	1-150	$2.58\cdot10^{3} \pm 1.36\cdot10^{2}$	$4.91\cdot10^{3} \pm 9.84\cdot10^{3}$	$7.69\cdot10^{3}$	0.9972	49	0.16
4-NP	2:1	Urine	1-150	$9.29\cdot10^{2} \pm 7.40\cdot10$	$1.13\cdot10^{3} \pm 5.35\cdot10^{3}$	$4.18\cdot10^{3}$	0.9937	14	0.16
		Wastewater	1-150	$1.52\cdot10^{3} \pm 5.88\cdot10$	$-1.12\cdot10^{3} \pm 4.25\cdot10^{3}$	$3.32\cdot10^{3}$	0.9985	24	0.17
		Sea water	1-150	$2.14\cdot10^{3} \pm 1.90\cdot10^{2}$	$1.38\cdot10^{4} \pm 1.37\cdot10^{4}$	$1.07\cdot10^{4}$	0.9921	34	0.24
	1:1	Urine	1-150	$1.13\cdot10^{3} \pm 3.07\cdot10$	$9.63\cdot10^{2} \pm 2.22\cdot10^{3}$	$1.73\cdot10^{3}$	0.9993	18	0.17
		Wastewater	1-150	$4.15\cdot10^{3} \pm 2.16\cdot10^{2}$	$-1.39\cdot10^{4} \pm 1.56\cdot10^{4}$	$1.22\cdot10^{4}$	0.9973	61	0.18
		Sea water	1-150	$2.32\cdot10^{3} \pm 1.22\cdot10^{2}$	$2.66\cdot10^{3} \pm 8.84\cdot10^{3}$	$6.91\cdot10^{3}$	0.9972	34	0.16
DHP	2:1	Urine	1-150	$2.41\cdot10^{4} \pm 2.18\cdot10^{3}$	$-1.38\cdot10^{5} \pm 1.58\cdot10^{5}$	$1.23\cdot10^{5}$	0.9918	22	0.21

	Wastewater	1-150	$5.89 \cdot 10^4 \pm 3.07 \cdot 10^3$	$1.33 \cdot 10^5 \pm 2.22 \cdot 10^5$	$1.73 \cdot 10^5$	0.9973	71	0.18
	Sea water	1-150	$1.06 \cdot 10^5 \pm 9.70 \cdot 10^3$	$-2.74 \cdot 10^4 \pm 7.01 \cdot 10^5$	$5.48 \cdot 10^5$	0.9917	117	0.27
	Urine	1-150	$3.91 \cdot 10^4 \pm 2.62 \cdot 10^3$	$1.08 \cdot 10^5 \pm 1.89 \cdot 10^5$	$1.48 \cdot 10^5$	0.9955	46	0.17
1:1	Wastewater	1-150	$1.27 \cdot 10^5 \pm 1.15 \cdot 10^4$	$8.72 \cdot 10^5 \pm 8.31 \cdot 10^5$	$6.49 \cdot 10^5$	0.9918	165	0.19
	Sea water	1-150	$1.67 \cdot 10^5 \pm 1.33 \cdot 10^4$	$-2.61 \cdot 10^5 \pm 9.60 \cdot 10^5$	$7.50 \cdot 10^5$	0.9937	191	0.16

b: slope; s_b: standard deviation of the slope; a: intercept; s_a: standard deviation of the intercept; $s_{y/x}$: standard deviation of the estimate; R^2: determination coefficient; ME: matrix effect.

Table S11. Toxicological data of the individual components of the eutectic mixtures used for the extraction of the target analytes.

Components of the eutectic mixture	Amount of each component per extraction for 1:1 molar ratio	Amount of each component per extraction for 2:1 molar ratio	Hazard classification and labelling	Risk and safety statements	Environmental toxicity	References
Fenchol	65.3 mg	130.6 mg	GHS07: Irritant	R 36/37/38<br>S 22-24/25-26	Not classified as a persistent, bioaccumulative and toxic in the environment	1<br>2
Acetic acid	24.2 µL	24.2 µL	GHS02: Flammable<br>GHS05: Corrosive	R 10-35<br>S 23-26-45	Not classified as a persistent, bioaccumulative and toxic in the environment	3<br>4

[1] Data taken from Sigma-Aldrich (chrome-extension://efaidnbmnnnibpcajpcglclefindmkaj/https://www.sigmaaldrich.com/ES/es/sds/aldrich/196444).

[2] Data taken from ChemWhat (https://www.chemwhat.pt/fenchol-cas-1632-73-1/).

[3] Data taken from Sigma-Aldrich (chrome-extension://efaidnbmnnnibpcajpcglclefindmkaj/https://www.sigmaaldrich.com/ES/en/sds/sial/537020).

[4] Data taken from International Labour Organization (https://www.ilo.org/legacy/english/protection/safework/cis/products/safetytm/clasann4.htm).

Risk and safety statements:

R10: Flammable.

R35: Causes severe burns.

R36/37/38: Irritating to eyes, skin, and respiratory system.

S22: Do not breathe dust.

S23: Do not breathe vapor.

S24/25: Avoid contact with eyes and skin.

S26: In case of contact with eyes, rinse immediately with abundant water and seek medical advice.

S45: In case of accident or if you feel unwell seek medical advice immediately and show the label.

Table S12. Comparison of previous works in which common analytes with this work have been extracted using DLLME procedures.

Extractant solvent	Analytes	Sample (volume)	Extraction technique (extractant volume) (dispersant volume)	Separation and determination technique	Recovery % (RSD %)	LOQs method	Comments	Reference
DL-menthol:dodecanoic acid DES (3:1 molar ratio)	BPF and BPA	Green and red tea infusions (5 mL)	AA-DLLME-SFO (100 µL) (-)	HPLC-UV	84-97 (1-5)	2.5 µg/L	BPB and 5 PAHs were also determined.	Food Chem. 2021, 348, 129106
Menthol:acetic acid DES (1:1 molar ratio)	DBP and BBP	Soft drinks and infusions (20 mL)	DLLME-SFO (100 µL) (-)	HPLC-UV	92-123 (1-14)	3.5-5.6 µg/L	A total of 9 PAEs were determined.	J. Chromatogr. A 2021, 1646, 462132
Octanoic acid:1-dodecanol DES (1:3 molar ratio)	BPF, BPA and 17β-estradiol	Sewage (4 mL)	VA-DLLME-SFO (80 µL) (-)	HPLC-FD	85-112 (1-5)	0.00505-0.00964 µg/L	Estriol was also determined.	Microchem. J. 2021, 163, 105915
C_9:C_{10}:C_{12} DES (1:1:1 molar ratio)	BPA and 4-NP	Tap and river water (5 mL)	AA-DLLME-SFO (200 µL) (-)	HPLC-PDA	91-104 (4-29)	5.00-7.02 µg/L	A total of 5 EDCs were determined.	J. Chromatogr. A 2020, 1629, 461498
Chloroform	BPA, DBP and BBP	Sea water (10 mL)	DLLME (125 µL) (1000 µL)	GC-MS	97 (6-10)	0.025-0.25 µg/L	A total of 54 environmental pollutants were determined. EtOH was used as disperser solvent.	Chemosphere 2022, 286, 131588
Chloroform	Estrone, 17β-estradiol, BPA, 4-OP and 4-NP	Chicken, fish and aquaculture pond water (6 mL)	DLLME (83 µL) (994000 µL)	HPLC-MS	88-109 (2-9)	0.07-0.23 µg/L	Estriol was also determined. Acetone was used as disperser solvent.	Food Chem. 2016, 192, 98-106
[BMIM][Cl]	BPA, 4-tOP, 4-OP and 4-NP	Sea water and industrial effluent	VA-DLLME (38 µL) (-)	HPLC-DAD	68-111 (-)	33.3-290 µg/L	4-CP and 4-tBP were also determined. $LiNTf_2$ was added to promote a metathesis reaction.	Microchim. Acta 2011, 174, 213

		(10 mL)						
2-dodecanol	Testosterone	River and tap water (5 mL)	DLLME-SFO (50 µL) (231 µL)	HPLC-DAD	98-106 (1-6)	6.66 µg/L	A total of 9 steroids were determined. TBABr:acetic acid DES was used as dispersant.	Microchem. J. 2019, 149, 103988
Chloroform	17β-estradiol and estrone	Mineral, run-off and waste water (7.5 mL)	VA-DLLME (500 µL) (110 µL)	CE-MS	56-91 (2-18)	1.27-3.07 µg/L	A total of 12 estrogens were determined. ACN was used as dispersant.	J. Chromatogr. A 2014, 1344, 109-121
Octanoic acid	Estrone and 17β-estradiol	Human urine (0.1 mL)	VA-DLLME (20 µL) (70 µL)	HPLC-MS/MS	83-120 (2-15)*	0.03-0.33 µg/L	Estriol and EE2 were also determined. HFIP was used as a dispersing solvent. The mixture between octanoic acid and HFIP formed a SUPRAS during DLLME.	J. Chromatogr. A 2018, 1580, 12-21
[C_6MIM][PF_6]	Estrone and 17β-estradiol	River, sea and waste water (5 mL)	DLLME (20 µL) (1000 µL)	HPLC-DAD	89-105 (4-6)	0.33-1.67 µg/L	A total of 5 estrogens were determined. Acetone was used as disperser solvent.	Anal. Lett. 2012, 45, 14, 1995-2005
1-dodecanol	BPA and gemfibrozil	Stream, well and waste water (10 mL)	DLLME (100 µL) (500 µL)	HPLC-MS/MS	119-120 (1-9)	0.5 µg/L	The method was also applied to the extraction of pesticides, and other PCPs. MeOH was used as disperser solvent.	Microchim. Acta 2015, 182, 1765-1774
**Fenchol:acetic acid DES (2:1 and 1:1 molar ratios)**	**Gemfibrozil, BPF, BPA, 17β-estradiol, testosterone, estrone, levonorgestrel, 4-tOP, BBP, DBP, 4-OP, 4-NP and DHP**	**Human urine, waste water and sea water (20 mL)**	**DLLME-SFO (100 µL DES for 2:1 and 90 µL for 1:1) (-)**	**HPLC-UV and UHPLC-MS/MS**	**43-100 (2-16) for 2:1 and 39-99 (2-19) for 1:1**	**0.130-0.385 µg/L for 2:1 and 0.122-0.293 µg/L for 1:1**	**-**	**This work**

*Value for all the analytes studied.

4-CP: 4-cumylphenol; 4-NP: 4-nonylphenol; 4-OP: 4-octylphenol; 4-tBP: 4-tert-butylphenol; 4-tOP: 4-tert-octylphenol; AA: air-assisted; ACN: acetonitrile; BBP: benzylbutyl phthalate; BMIM: 1-butyl-3-methylimidazolium; BPA: bisphenol A; BPB: bisphenol B; BPF: bisphenol F; C_6MIM: 1-hexyl-3-methylimidazolium; CE: capillary electrophoresis; DAD: diode array detector; DBP: dibutyl phthalate; DES: deep eutectic solvent; DLLME: dispersive liquid-liquid microextraction; EDC: endocrine disrupting chemical; EE2: 17α-ethinylestradiol; EtOH: ethanol; FD: fluorescence detector; GC: gas chromatography; HFIP: hexafluoroisopropanol; HPLC: high-performance

liquid chromatography; $LiNTf_2$: lithium bis[(trifluoromethane)sulfonyl]imide; LOQ: limit of quantification; MeOH: methanol; MS/MS: tandem mass spectrometry; MS: mass spectrometry; PAE: phthalic acid ester; PAH: polycyclic aromatic hydrocarbon; PDA: photo diode array; PF_6: hexafluorophosphate; PCP: personal care product; RSD: relative standard deviation; SFO: solidification of floating organic drop; SUPRAS: supramolecular solvent; TBABr: tetrabutylammonium bromide; UHPLC: ultra-high-performance liquid chromatography; UV: ultraviolet; VA: vortex-assisted.

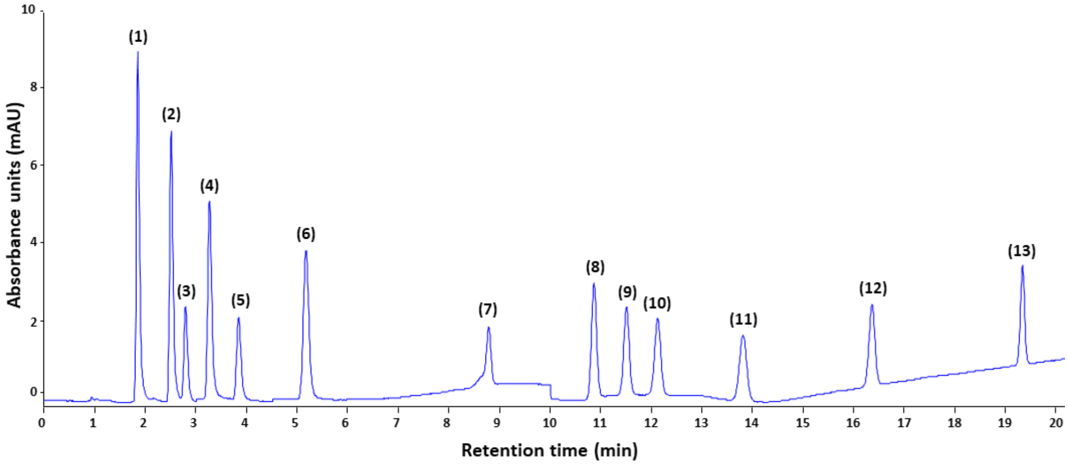

Figure S1. HPLC-UV chromatogram of a working solution of the analytes in ACN:water (50:50, v/v). Column: Eclipse Plus C_{18} (10 cm x 4.6 mm, 3.5 µm). Injection volume: 20 µL. Flow rate: 1.0 mL/min. Separation at 40 ºC. Concentration of all the analytes: 500 µg/L. Peak identification: BPF (1), BPA (2), 17β-estradiol (3), testosterone (4), estrone (5), levonorgestrel (6), gemfibrozil (7), 4-tOP (8), BBP (9), DBP (10), 4-OP (11), 4-NP (12), DHP (13).

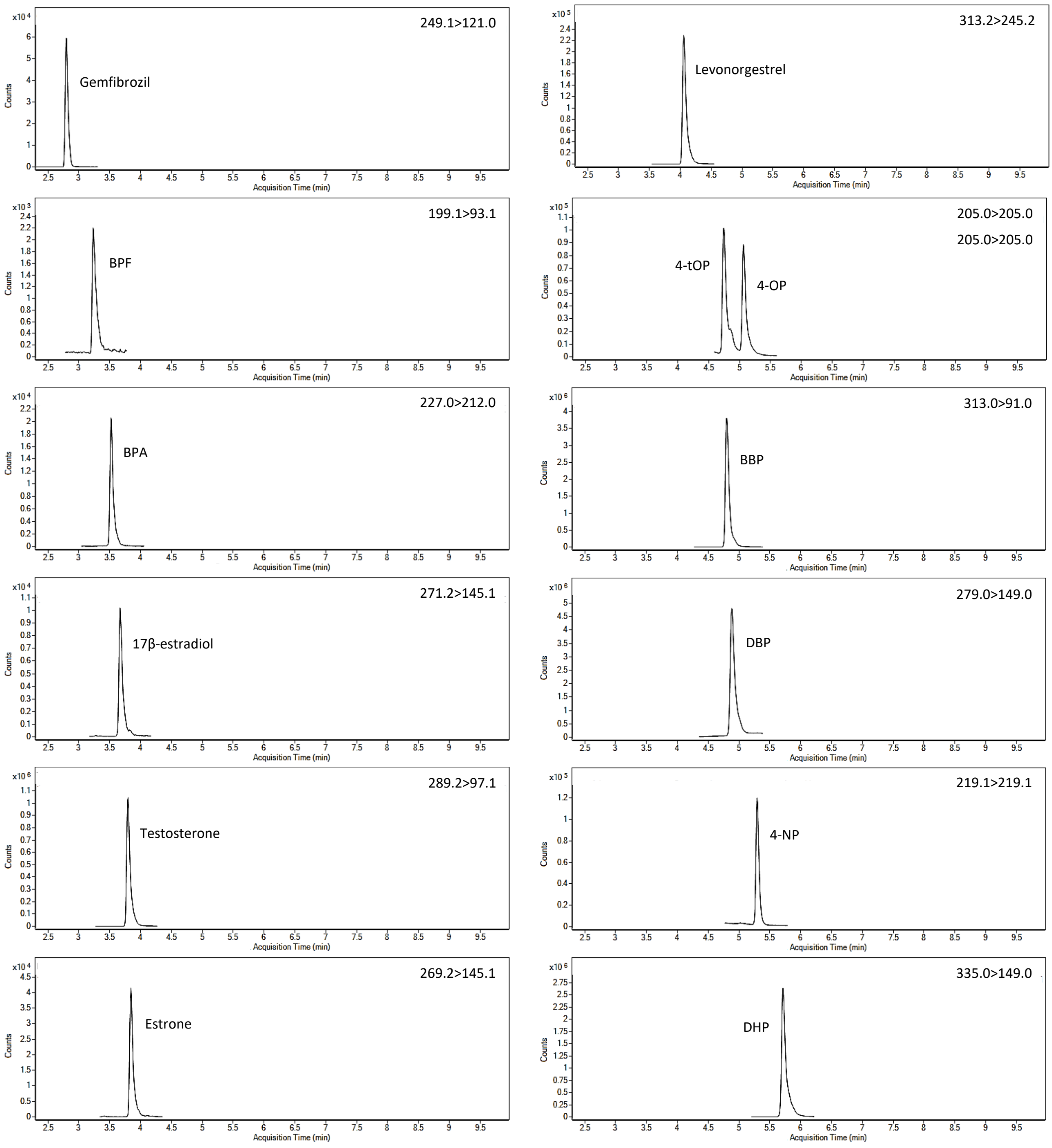

Figure S2. UHPLC-MS/MS dynamic MRM chromatogram of a wastewater sample spiked with the target analytes at 100 µg/L, except for 17β-estradiol and estrone which was 150 µg/L. Extraction conditions: 20 mL of sample at pH 6.0 and 25 ºC, 90 µL of eutectic mixture at 1:1 molar ratio, and manual agitation for 1 min. Final extract was dissolved in 2310 µL ACN:water (50:50, v/v).

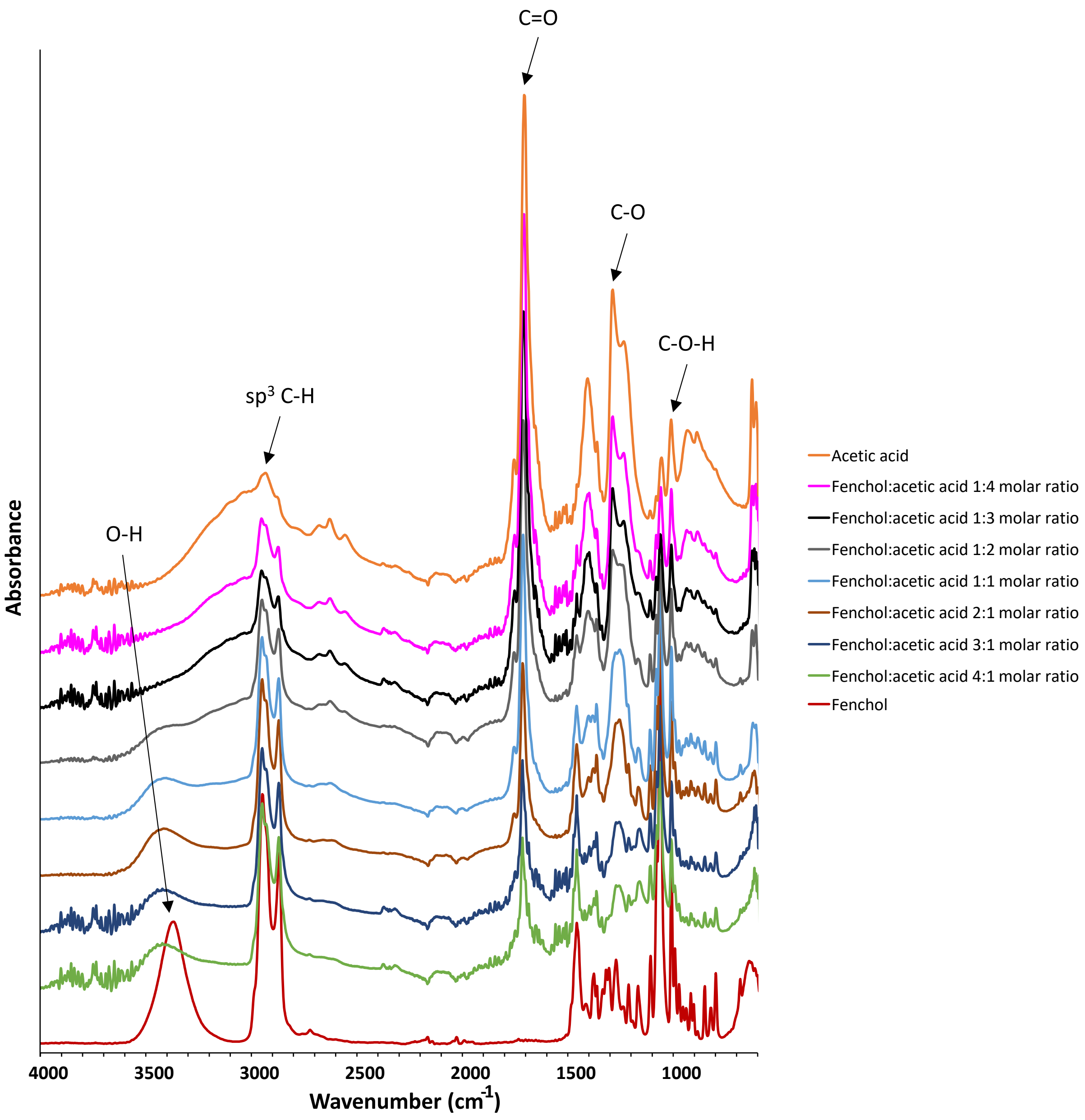

Figure S3. ATR-FTIR spectra of fenchol, acetic acid and the eutectic mixtures.

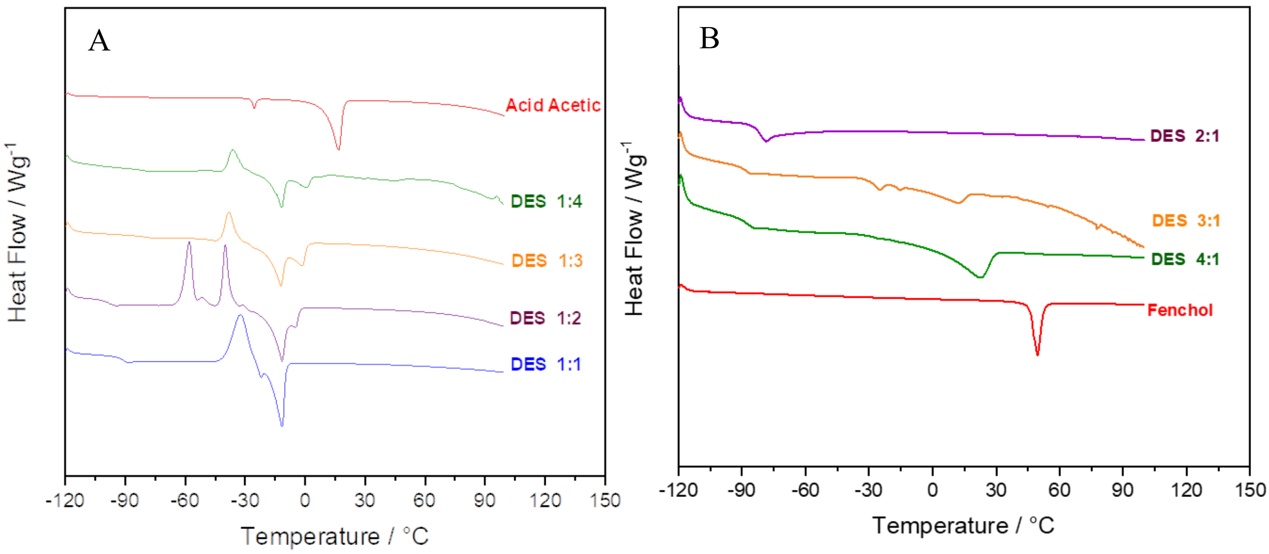

Figure S4. DSC curves obtained for all the eutectic mixtures. A) Acetic acid as the main component and B) Fenchol as the main component. Negative peaks represent endothermic processes.

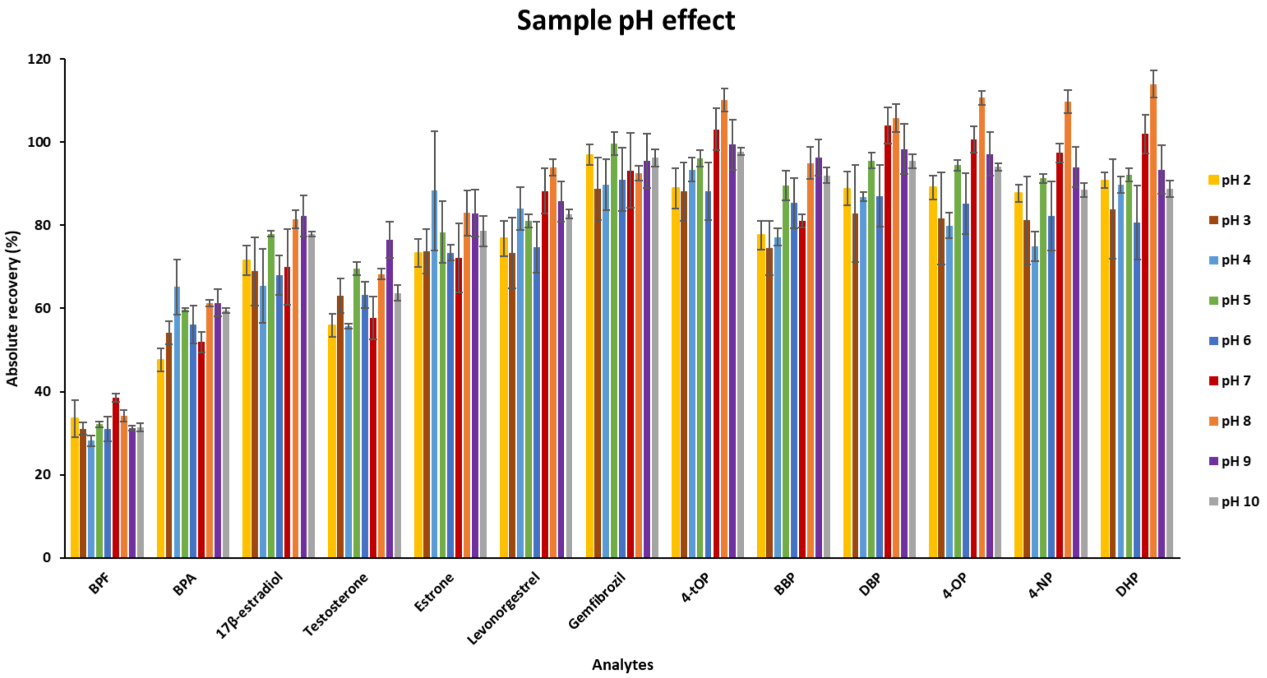

Figure S5. Effect of the sample pH on the absolute recovery values of the selected emerging contaminants using fenchol:acetic acid at 1:2 molar ratio as DLLME-SFO solvents. Extraction conditions: 20 mL of spiked Milli-Q water at 0.50 mg/L, except for 17β-estradiol and estrone which was 0.75 mg/L, at 25 ºC, 100 µL of eutectic mixture fenchol:acetic acid at 1:2 molar ratio, and manual agitation for 1 min.

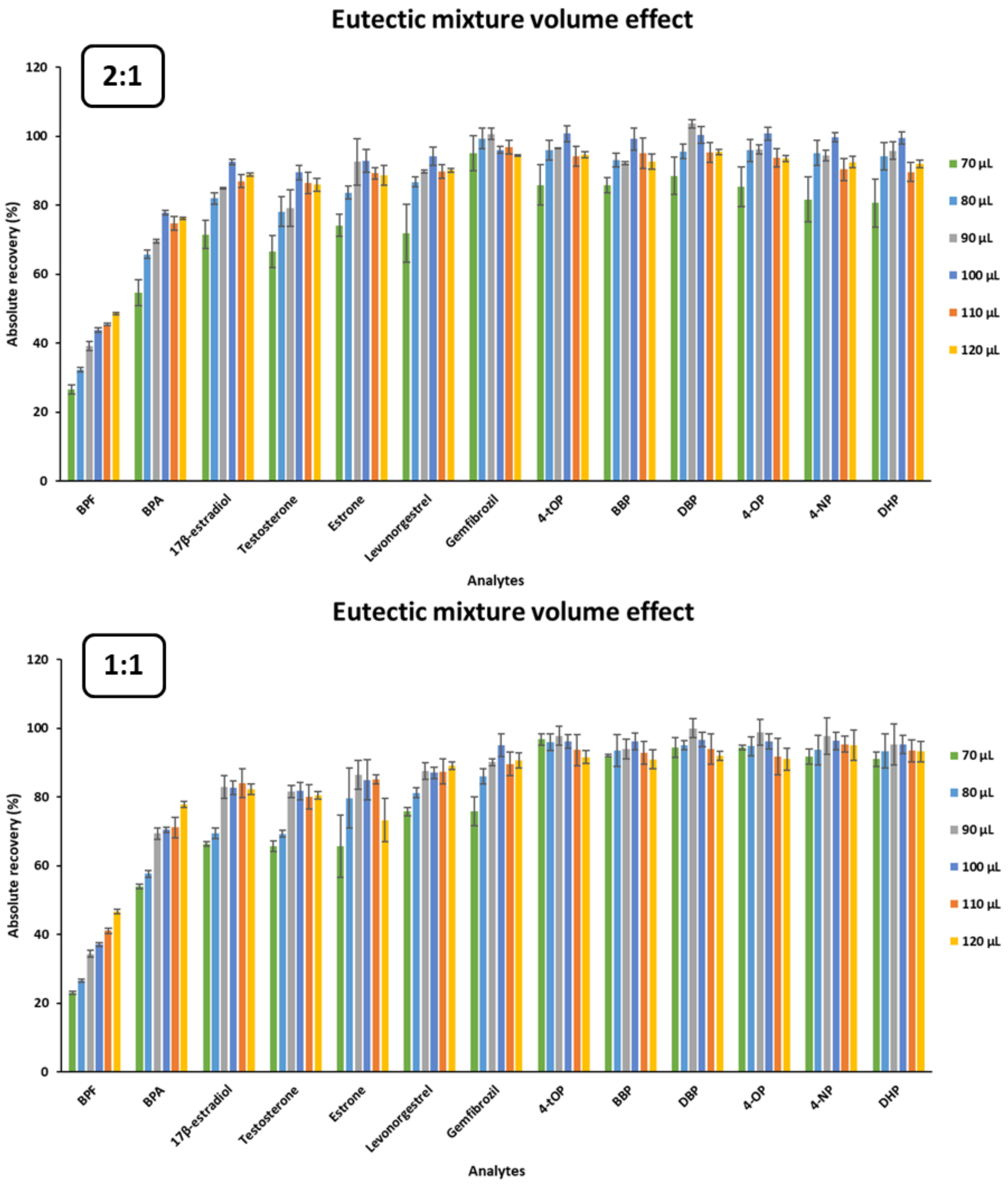

Figure S6. Effect of the eutectic mixture volume during the extraction step on the absolute recovery values of the selected emerging contaminants using 2:1 and 1:1 molar ratio mixtures of fenchol:acetic acid as DLLME-SFO solvents. Extraction conditions: 20 mL of spiked Milli-Q water at 0.50 mg/L, except for 17β-estradiol and estrone which was 0.75 mg/L, at pH 6.0 and 25 ºC, and manual agitation for 1 min.

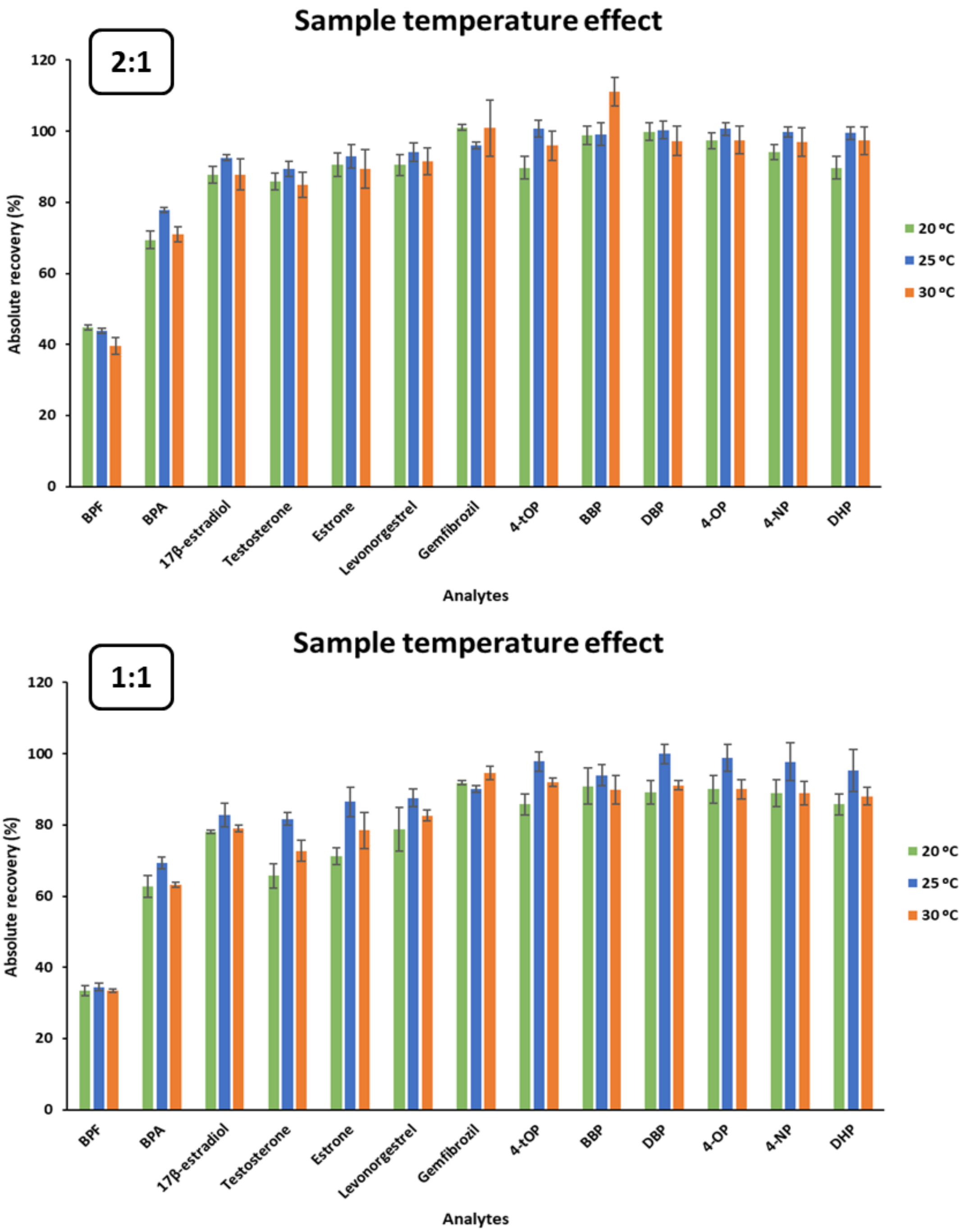

Figure S7. Effect of the extraction temperature during the extraction step on the absolute recovery values of the selected emerging contaminants using 2:1 and 1:1 molar ratio mixtures of fenchol:acetic acid as DLLME-SFO solvents. Extraction conditions: 20 mL of spiked Milli-Q water at 0.50 mg/L, except for 17β-estradiol and estrone which was 0.75 mg/L, at pH 6.0, 100 µL of eutectic mixture at 2:1 molar ratio and 90 µL for the one at 1:1 molar ratio, and manual agitation for 1 min.
